# High Throughput Fluorescence-Based In Vitro Experimental Platform for the Identification of Effective Therapies to Overcome Tumour Microenvironment-Mediated Drug Resistance in AML

**DOI:** 10.3390/cancers15071988

**Published:** 2023-03-27

**Authors:** Yoana Arroyo-Berdugo, Maria Sendino, David Greaves, Natalia Nojszewska, Orest Idilli, Chi Wai So, Lucy Di Silvio, Ruby Quartey-Papafio, Farzin Farzaneh, Jose Antonio Rodriguez, Yolanda Calle

**Affiliations:** 1 School of Health and Life Sciences, University of Roehampton, London SW15 4JD, UK; 2 Department of Genetics, Physical Anthropology and Animal Physiology, University of the Basque Country (UPV/EHU), 48940 Leioa, Spain; 3 Department of Haemato-Oncology, King’s College London, London SE5 9NU, UK; 4 Faculty of Dentistry, Oral & Craniofacial Sciences, King’s College London, London SE1 9RT, UK

**Keywords:** AML, tumour microenvironment, co-culture system, daunorubicin, resistance, KPT-330, selinexor

## Abstract

**Simple Summary:**

The interactions between Acute Myeloid Leukaemia (AML) cells and the surrounding bone marrow (BM) tissue contribute to blocking the efficacy of current drug treatments and to the relapse of patients. Relapsing AML tumours are refractory to current therapies and remain untreatable. Developing new therapies for AML requires the development of new drug-screening methods using in vitro models that closely mimic the interactions of AML cells with cytoprotective BM cells. We have developed a new fluorescence-based in vitro model and an analytical method that takes into consideration the reciprocal interactions between AML cells and the protective BM stroma during drug treatments. Using this new method and combining it with bioinformatics, we have identified new combinations of drugs that may overcome resistance to drug treatments and lead to improved therapies for AML.

**Abstract:**

The interactions between Acute Myeloid Leukaemia (AML) leukemic stem cells and the bone marrow (BM) microenvironment play a critical role during AML progression and resistance to drug treatments. Therefore, the identification of novel therapies requires drug-screening methods using in vitro co-culture models that closely recreate the cytoprotective BM setting. We have developed a new fluorescence-based in vitro co-culture system scalable to high throughput for measuring the concomitant effect of drugs on AML cells and the cytoprotective BM microenvironment. eGFP-expressing AML cells are co-cultured in direct contact with mCherry-expressing BM stromal cells for the accurate assessment of proliferation, viability, and signaling in both cell types. This model identified several efficacious compounds that overcome BM stroma-mediated drug resistance against daunorubicin, including the chromosome region maintenance 1 (CRM1/XPO1) inhibitor KPT-330. In silico analysis of genes co-expressed with CRM1, combined with in vitro experiments using our new methodology, also indicates that the combination of KPT-330 with the AURKA pharmacological inhibitor alisertib circumvents the cytoprotection of AML cells mediated by the BM stroma. This new experimental model and analysis provide a more precise screening method for developing improved therapeutics targeting AML cells within the cytoprotective BM microenvironment.

## 1. Introduction

Although 70–80% of Acute Myeloid Leukaemia (AML) patients undergo complete remission after chemotherapy [1,2] long-term disease-free survival remains at low levels (between 30–50% [1,2,3]). Therefore, there is an urgent, unmet need for improved therapies against AML. Devising new therapeutic interventions will require a better understanding of the tumour biology in AML that leads to relapse and drug resistance.

Currently, treatments for AML patients fit for intensive or low-intensive chemotherapy regimens are frequently based on the combination of daunorubicin and cytarabine [4,5,6]. These initial treatments can be followed by stem-cell transplantation [7]. Some more specialized therapies for specific AML subgroups may include dasatinib [8] and the FLT3 kinase inhibitor for FLT3-ITD AML patients [9]. Despite the improvements during the last decade in management of AML with the current therapies, the majority of AML patients will relapse within 3 years [4]. A relapse in AML has been ascribed to minimal residual disease (MRD) in the bone marrow (BM) [3,10,11], and there is compelling evidence that the adhesive interactions between AML stem cells and the BM niches are vital in MRD, leading to a resistance to drug treatments [3,12] as well as in AML maintenance and progression [13,14].

Given the emerging key role of the BM microenvironment in AML, the identification of new therapeutic interventions to overcome cell adhesion-mediated drug resistance (CAM-DR) requires the development of in vitro co-culture models that recreate the in vivo BM setting for studies of the biology of AML, as well as for drug screening [13]. Despite some advances using primary samples from AML patients [15] or luciferase-based technology and AML cell lines [16], currently available in vitro co-culture systems fail to discriminate and evaluate the concomitant impact of drugs on AML cells and other BM cells. It is becoming evident that understanding the direct impact of drug treatments on the tumour microenvironment in the presence of cancer cells is critical to accurately evaluate drug efficacy against cancer cells. Therapeutic drugs can induce direct cancer cell killing while simultaneously stimulating the tumour microenvironment to paradoxically induce the formation of niches that promote the resistance of cancer cells against treatments [17,18,19]. Determining the concomitant effect of drug treatments in cancer and tissue stromal cells can lead to new strategies for drug discovery. Preclinical models that measure the simultaneous effect of drugs on both stromal and tumour cells are, therefore, better predictors of therapeutic drug efficacy [19].

We have previously devised a fluorescence-based experimental model for drug screening against multiple myeloma (MM) that is scalable to high throughput. This model provides a significant improvement on previous methodologies [20] by allowing for the assessment of both MM and BM stromal responses (BM fibroblastic cells and osteoclasts) to anticancer drugs [19]. Using this methodology, we revealed that a resistance to dexamethasone (a MM drug already in use in the clinic with a widespread problem of resistance being developed over time) involves the enhancement of stromal cell proliferation in response to the drug treatment [19], as well as the previously described MM cell attachment to stromal cells. We routinely use this platform in our current drug discovery projects.

We now report a new fluorescence-based in vitro co-culture system-based high throughput analysis for the assessment of drug efficacy against AML. This AML experimental model is based on the same principles as our MM method. For AML, we tested the interactions of eGFP–AML-expressing cells with the BM mesenchymal fibroblastic cell line mCherry-HS5 and primary osteoblasts, as both BM cell types have been shown to provide cytoprotection against drug treatments in AML [21,22,23].

Using our new platform, we found distinctive patterns of interaction between AML and BM mesenchymal/fibroblastic stromal cells that differ from the interactions of MM cells with the same BM niche. We also performed a drug screening and identified some pharmacological candidates that overcome BM-mediated resistance to daunorubicin, including KPT-330 (selinexor, xpovio^®^, nexpovio^®^), an inhibitor of the nuclear export receptor CRM1/XPO1. By combining in silico analysis using transcriptomics data of AML patients with in vitro testing using our new AML experimental model, we also identified the potentially efficacious combination of KPT-330 with the AURKA pharmacological inhibitor alisertib to prevent CAM-DR. Our data warrant further investigation of these identified drug combinations in order to improve the efficacy of AML therapy.

## 2. Materials and Methods

### 2.1. Cell Culture

The Human AML cell lines eGFP-MOLM-14, eGFP-MV4-11, eGFP-Kasumi, and eGFP-THP-1 were generated using lentiviral vectors, as previously described [19]. The BM mesenchymal/fibroblastic stromal cell line mCherry-HS5 cells were previously characterized [19]. All cell lines were cultured at 37 °C in a humidified atmosphere in the presence of 5% CO_2_, 95% air. AML cells were cultured in RPMI-1640 medium and mCherry-HS5 cells in DMEM supplemented with L-glutamax and 10% foetal bovine serum (FBS). Primary Human osteoblasts were obtained from Lonza Ltd. and were grown using the OGM™ Osteoblast Growth Mediu BulletKit™.

### 2.2. Determination of Cell Proliferation in eGFP-AML Cell Lines in Co-Culture with mCherry-HS5 Cells

mCherry-HS5 BM fibroblastic stromal cells were seeded at 10 × 10^3^ cells per well in 96 well plates and incubated overnight in DMEM supplemented with 10% FBS. The following day, the culture media was aspirated and eGFP–AML cells were layered on mCherry-HS5 cells at a density of 2 × 10^5^ cells/mL in 200 μL per well of RPMI supplemented with 10% FBS. Three technical replicas were seeded per experimental condition. The library of compounds tested in our high throughput studies was Library I-384 from Merck. To determine the proliferation index, the fluorescence intensity (FI) per well was read at λex488 nm/λem528 nm and at λex584 nm/λem607 nm to estimate the numbers of eGFP-AML cells and mCherry-HS5 cells, respectively, using a FLx800 multidetection microplate reader (Biotek Instruments, Winooski, VT, USA). Measurements were taken at d0 and d3 and the proliferation index was calculated as the ratio of the fluorescence emission at d3/d0 after subtracting the background emission. Each experiment was repeated three times. Quest Graph™ EC50 Calculator, AAT Bioquest, Inc., https://www.aatbio.com/tools/ec50-calculator (accessed on 15 February 2023) was used to calculate the half maximal inhibitory concentration (IC50) and the half maximal efficacious concentration (EC50) values.

### 2.3. Determination of AML Cell Viability by Flow Cytometry

The percentage of viable and apoptotic cells was determined using flow cytometry. Cell cultures were harvested and Annexin-V-APC and propidium iodide staining in the eGFP positive population (AML cells) was measured by flow cytometry using a BD FACSCanto II flow cytometer (BD BioSciences, Franklin Lakes, NJ, USA) equipped with a High Throughput Sampler.

### 2.4. Cell Cycle Analysis

The percentage of cells in the different phases of the cell cycle was determined according to the DNA content using the propidium iodide staining detected by FACS as a readout. Cells seeded in 96 well plates were fixed with cold 70% ethanol for 30 min. Cells were washed twice with PBS and then resuspended in 50 μL of PBS with propidium iodide (50 μg/mL) and RNase (100 μg/mL) and incubated in the dark for 30 min. The levels of propidium iodide staining were detected using a BD FACSCanto II flow cytometer (BD BioSciences, Franklin Lakes, NJ, USA).

### 2.5. In Silico Analysis of the Correlation between the Expression of CRM1-Encoding XPO1 Gene and Genes in the TARGET Database

The potential co-expression of *XPO1* (the gene encoding CRM1) with the 135 genes collected in the CGA TARGET (Tumour Alterations Relevant for GEnomics-driven Therapy) database of the Broad Institute (https://software.broadinstitute.org/cancer/cga/target (accessed on 11 January 2019)) (herein referred to as TARGET genes) was performed using two previously described bioinformatics tools. CANCERTOOL [24] was used for the analysis of breast, lung, prostate, and colorectal cancer samples, and the cBioPortal analysis suite [25,26] was used for the analysis of AML samples. Pearson’s correlation values (R) were determined for each *XPO1*/TARGET gene pair in each tumor type analyzed, and the mean R value across the five tumor types was calculated and used as a criterion to select *XPO1*-co-expressed genes. Significant co-expression correlation values (*p* < 0.05) higher than 0.2 or lower than −0.2 were considered to calculate an average correlation value for the five types of cancer for each gene from the CGA TARGET database.

### 2.6. Statistics

For statistical analysis using the adequate tests, GraphPad Prism 9 software was used. Statistically significant difference using ANOVA or Student *t*-test was determined from *p* < 0.05. The interaction of pairs of pharmacological compounds under study was analyzed using Calcusyn software (Biosoft, Ferguson, MO, USA) based on the Chou–Talalay method [27]. Data from the percentage of apoptotic cells were expressed as the fraction of affected cells by the treatments (Fa) by the compounds as single agents or in combination with respect to untreated cells to calculate the combination index (CI) of the compounds CI ≤ 1 and CI = 1 indicate synergism and additive effects, respectively.

## 3. Results

### 3.1. Characterisation of a High Throughput Experimental Platform to Distinguish Changes in the Proliferation of AML and BM Mesenchymal Stromal Cells in Co-Culture

Methods such as the MTT assay, commonly used to determine the numbers of cells in the culture, are unable to differentiate the presence of stromal and AML cells when in co-culture. Previous AML studies have used luciferase-expressing AML cells to distinguish the proliferative response of AML in co-culture with BM stromal cells [16]. However, to our knowledge, there are no published methods to determine simultaneously the reciprocal effect of AML cells and/or anticancer therapy on the stromal cell compartment in vitro and in a high throughput setting. We used our previously published approach [19] to develop a new fluorescence-based high throughput platform to determine the inter-relation between AML and BM stromal niches and the response to drug treatments.

**Figure 1:**
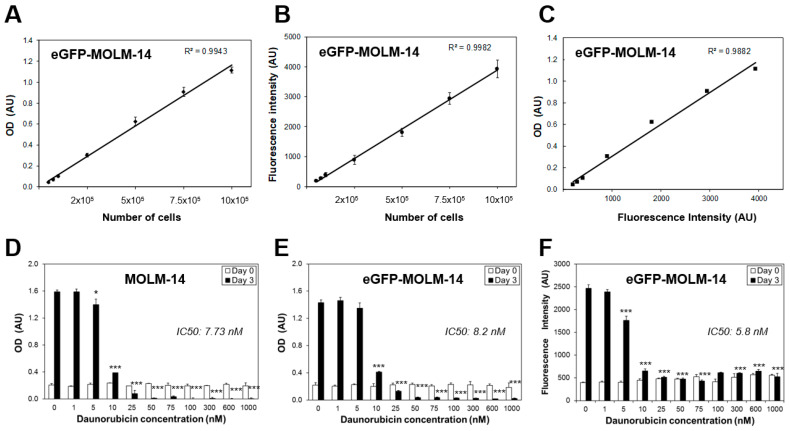
Characterization of eGFP-expressing AML cells. Comparison of the sensitivity of estimation of cell numbers by measuring optical density (OD) using an MTT assay in arbitrary units (AU), or fluorescent intensity emitted by eGFP-expressing tumour cells. Correlation between values of cell numbers seeded per well and OD (**A**) or fluorescent intensity (**B**). Correlation of values of OD and fluorescent intensity (**C**). Proliferation of parental (**D**) and eGFP-expressing MOLM-14 cells (**E**) left untreated or treated with increasing concentrations of daunorubicin was estimated using MTT assays or fluorimetry (**F**). The half maximal inhibitory concentration (IC50) values for inhibition of the proliferation of the cell lines are shown in the graphs. Plates used for measurements of optical density were previously used to measure fluorescent intensity of the cultures of eGFP-MOLM-14. * *p* < 0.05; *** *p* < 0.005 ANOVA test versus control untreated.

The AML cell lines MOLM-14, MV4-11, Kasumi, and THP-1 were chosen for assessment in our model as they represent some of the most common genetic and cytogenetic abnormalities observed in AML patients, including those poor prognostic subtypes carrying FLT3 internal tandem duplication [28] and MLL-gene fusions [29], as well as for representing different possible differentiation status of AML blasts. We generated eGFP-expressing versions of these cell lines so that the fluorescent signal emitted by the cells allowed for the determination of cell numbers in culture with comparable sensitivity to the MTT assay. The levels of expression of eGFP in the AML cell lines were equivalent to those of the previously generated MM cell lines [19]. Accordingly, eGFP-expressing AML cells showed linear correlations between the number of seeded cells and the emitted fluorescent signal with equivalent sensitivity to the MTT assay (Figure 1A–C and Figure S1). GFP-expressing cells maintained the same proliferation rates and the sensitivity to anticancer drugs, such as daunorubicin as the parental cell lines (Figure 1D–F and Figure S2).

The presence of mCherry-HS5 cells in co-culture did not alter the levels of expression of eGFP in eGFP–AML-viable cells (Figure S3). This shows that our determination of cell numbers based on integrated eGFP fluorescence intensity is applicable for eGFP–AML cells cultured alone or in the presence of other cell types. Non-viable eGFP–AML cells underwent a reduction in eGFP levels similar to those previously described in MM cells [19] (Figure S3C–F), so that the low-expressing cells are detectable by flow cytometry without interfering with the eGFP signal required to estimate viable eGFP cell numbers by fluorimetry [19].

**Figure 2:**
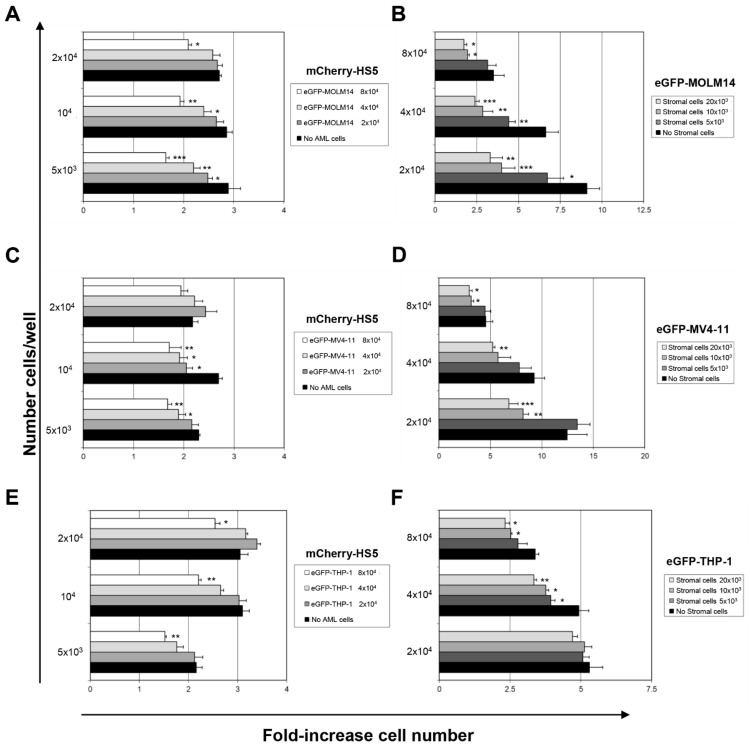
Analysis of stromal and tumour cells’ proliferation in co-culture. Fluorescent-based analysis of proliferation of mCherry-HS5 stromal cells (**A**,**C**,**E**) and eGFP-AML cell lines (**B**,**D**,**F**). Proliferation of cells was evaluated after 72 h. Histograms indicate the fold-increase in cell numbers. * *p* < 0.05; ** *p* < 0.01; *** *p* < 0.005; ANOVA test versus cells cultured alone.

We assessed the reciprocal influence on proliferation between mCherry-HS5 cells and eGFP-tumour cell lines (Figure 2) and found that the presence of AML cells affected the proliferation of mCherry-HS5 BM stromal cells in a distinctive manner that was different from the effect elicited by the presence of MM cells [19]. We previously found that MM cell lines significantly increased the proliferation rate of mCherry-HS5 cells seeded at low density (≤10 × 10^3^ mCherry-HS5 cells/well) [19]. Using the same cell densities as a starting point, we now report that AML cells either did not affect the proliferation of mCherry-HS5 cells or inhibited their proliferation when AML cells were seeded at a density of 4 × 10^4^ cells/well or above (Figure 2A,C,E).

The impact of the presence of mCherry-HS5 cells on AML cell proliferation presented some variations among the cell lines tested. Thus, the proliferation of eGFP-MOLM-14 cells was either not affected or halted by mCherry-HS5 cells (Figure 2B). eGFP-MV4-11 cells, on the other hand, presented a pattern of proliferation in response to the presence of mCherry-HS5 cells that varied depending on the initial AML cell density used. mCherry-HS5 cells increased the proliferation of eGFP-MV4-11 cells (Figure 2D) seeded at lower densities (≤5 × 10^3^ cells/well). However, when seeded at a density above 2 × 10^4^ cells/well, the co-culture with increasing numbers of mCherry-HS5 cells correlated with an inhibition of the proliferation of eGFP-MV4-11 cells (Figure 2D). The proliferation of eGFP-THP-1 cells was generally unaffected by the presence of mCherry-HS5 cells. Only when eGFP-THP-1 cells were seeded at the highest tested densities (≥4 × 10^4^ cells/well) was their expansion reduced by the presence of mCherry-HS5 cells (Figure 2F).

Overall, the presence of AML cells failed to stimulate the proliferation of mCherry-HS5 cells, while this BM stromal cell line did not promote the proliferation of sparsely seeded AML cells. These results are in contrast to the mCherry-HS5-mediated stimulation of the proliferation of MM cells that we previously reported [19]. Taken together, our results show that AML cells display distinctive heterotypic cell–cell interactions with mCherry-HS5 cells that differ from previous patterns of interaction by MM cells and other types of cancers that can colonize the BM [19].

**Figure 3:**
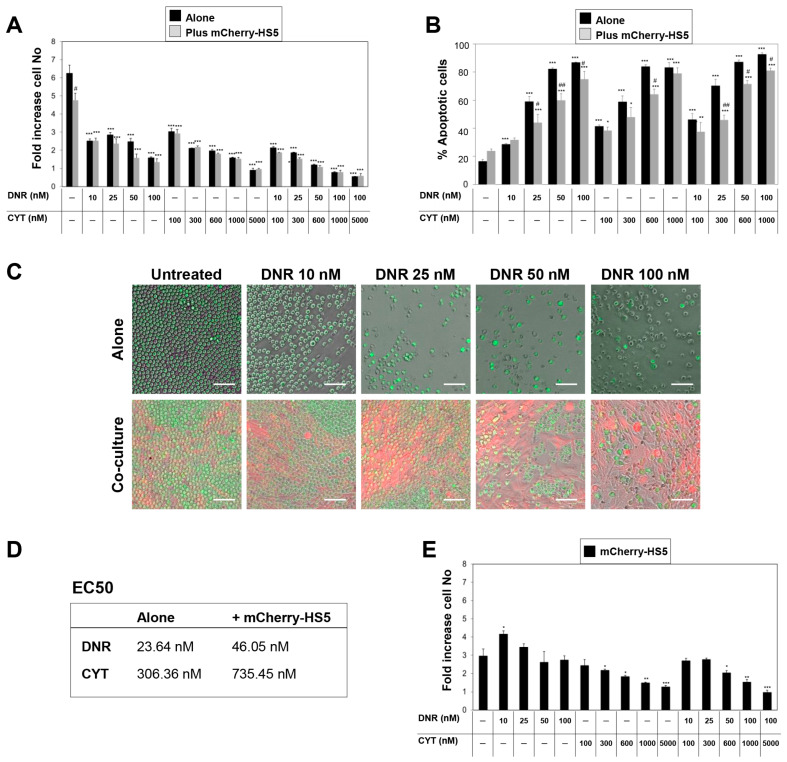
Analysis of the response to daunorubicin and cytarabine of eGFP–MOLM-14 cells cultured alone or in heterotypic cultures with mCherry-HS5 cells. Analysis of changes in proliferation (**A**) and percentage of apoptotic cells (**B**) in cultures of eGFP-MOLM-14 cells at Day 3 post-seeeding. Cultures were left or were treated with daunorubicin (DNR) or cytarabine (CYT) as single agents or in combination; (**C**) Composites of phase contrast and fluorescence micrographs of GFP-MOLM-14 cells (green) cultured alone or in co-culture with mCherry-HS5 cells (red) generated using NIS-Elements AR 5.10 software. Bar 40 μM; (**D**) Table showing the half maximal efficacious concentration (EC50) values of DNR and CYT for inducing apoptosis of eGFP-MOLM-14; (**E**) Proliferation of mCherry-HS5 cells. * *p* < 0.05; ** *p* < 0.01; *** *p* < 0.005 ANOVA test versus untreated cells under the same culture conditions. # *p* < 0.05; ## *p* < 0.01 post-hoc Student T-test comparing cells co-cultured with mCherry-HS5 cells vs. cells cultured alone treated with the same concentration of the drug under study.

We then used our methodology to analyze the capacity of mCherry-HS5 cells to inhibit the AML killing efficacy of daunorubicin and cytarabine at doses achievable in patients [30,31]. Treatment with these compounds inhibited the proliferation of AML cells in a concentration-dependent manner independently of the presence of BM stromal cells, and the combination of the two drugs significantly increased this anti-proliferative effect (Figure 3A). However, the analysis of the percentage of apoptotic cells showed that the presence of BM stromal cells inhibited the cytotoxic effect of daunorubicin and cytarabine, as previously reported [16] (Figure 3B,C), and increased the EC50 of both drugs (Figure 3D). The use of daunorubicin and cytarabine in combination did not prevent this cytoprotective effect (Figure 3B). Treatment of the heterotypic cultures with the lower concentrations of daunorubicin enhanced the proliferation of mCherry-HS5 cells and did not affect their proliferation at the higher concentrations used (Figure 3E). Cytarabine inhibited the proliferation of mCherry-HS5 cells in a concentration-dependent manner (Figure 3E). The BD Accuri flow cytometer used for analysis of the cells in co-culture lacked the adequate laser configuration to detect the protein mCherry. Hence, it was not possible to determine the exact percentage of apoptotic cells in the mCherry-HS5 population in our experiments. It remains to be elucidated whether the inhibition of mCherry-HS5 proliferation induced by cytarabine is due to a cytotoxic effect on these cells. Co-treatment with daunorubicin and cytarabine prevented the daunorubicin-induced increase in mCherry-HS5 proliferation (Figure 3E), but the remaining viable stromal cells sustained the cytoprotective effect against the drug combination (Figure 3B). Taken together, our data suggest that in the presence of BM mesenchymal/fibroblastic stromal cells, the inhibition of the proliferation of AML cells by daunorubicin and cytarabine is due to both cytostatic and cytotoxic effects.

**Figure 4:**
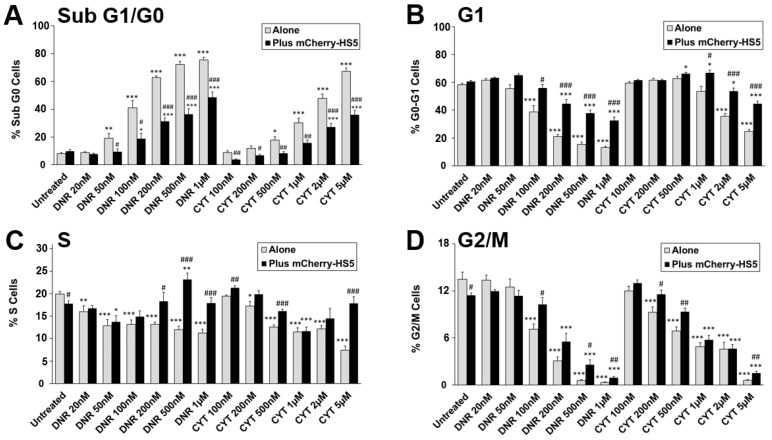
Analysis of percentage of eGFP-MV4-11 cells in each phase of the cell cycle cultured alone or in co-culture with mCherry-HS5 after drug exposure for 3 days. eGFP-MV4-11 cells cultured alone or in the presence of mCherry-HS5 cells were left untreated or were treated with daunorubicin (DNR) or cytarabine (CYT) for 3 days. Samples were ethanol-fixed and stained with propidium iodide for analysis of the cell cycle by FACS. Histograms represent mean values ± S.E. Percentage of cells in sub-G1/G0 phase (**A**), G1 (**B**), S (**C**) and G2/M (**D**) phases of the cell cycle. * *p* < 0.05, ** *p* < 0.01, *** *p* < 0.005 ANOVA versus control untreated in the same culture condition. # *p* < 0.05, ## *p* < 0.01, ### *p* < 0.005 post-hoc Student T-test cells co-cultured with mCherry-HS5 cells vs. cells cultured alone treated with the same concentration of drug treatment.

In order to further understand the mechanisms of cytoprotection against daunorubicin and cytarabine mediated by mCherry-HS5 cells, we analyzed the pattern of cell cycle progression of AML cells. Cell cycle analysis confirmed the cytoprotection mediated by the presence of mCherry-HS5 cells, resulting in a reduction in the percentage of late apoptotic (sub G1/G0 population) AML cells versus cells cultured alone (Figure 4A). The treatment of AML cells in monoculture with drugs as single agents induced a concentration-dependent decrease in the percentage of cells in all phases of the cell cycle (Figure 4B–D). In contrast, the presence of mCherry-HS5 blocked the efficacy of both drugs by inducing an accumulation of AML cells in S (Figure 4C) and G1 phases in comparison to AML cells cultured alone (Figure 4B). Additionally, a sustained higher percentage of mitotic cells (Figure 4D) was noted in the case of treatment with daunorubicin. Overall, these results indicate that when treated in monoculture, AML cells continue to cycle and drug treatments cause DNA damage in cells entering S phase, resulting in apoptosis and, therefore, a reduced rate of proliferating cells. However, when AML cells are treated in co-culture, the presence of BM stromal/fibroblastic cells induce a delayed transition from G1 and S phases, slowing down cell cycle progression and, hence, interfering with the mechanisms of action of daunorubicin and cytarabine during DNA synthesis, resulting in the inhibition of their pro-apoptotic effect.

Overall, these results show that our fluorescence-based experimental model provides insight for a better understanding of the behaviour of AML cells and BM mesenchymal/fibroblastic cells and the concomitant impact of drugs on both cell populations.

### 3.2. High Throughput Screening to Identify Drug Candidates and Signalling Pathways Involved in BM Stromal-Mediated Drug Resistance in AML

In MM, the presence of mCherry-HS5 cells provided a cytoprotective microenvironment against therapeutic drugs by promoting both the survival and proliferation of MM cells [19]. Our results so far indicate that in AML, mCherry-HS5 cells provide pro-survival signals but fail to sustain the proliferation of AML cells treated with daunorubicin and cytarabine. This implies that identifying candidate compounds that may prevent BM-stroma-mediated drug resistance requires the analysis of the percentage of cells undergoing apoptosis rather than the analysis of cell proliferation. This can be achieved by staining cells with Annexin V and propidium iodide as described in the above sections. We noticed that AML cells undergoing apoptosis (both early and late) can be clearly distinguished by flow cytometry by the low levels of expression of eGFP (Figure S4A). Our data using various AML cell lines consistently showed a clear correlation between the percentage of apoptotic cells determined by Annexin V staining and the percentage of GFP-low-expressing AML cells (Figure S4B,C). Hence, the quantification of the percentage of low eGFP-expressing cells is a valid and rapid method to determine the levels of apoptosis in GFP-AML cell cultures (Figure S4D,E). This novel approach eliminates several staining steps required in the standard protocol to determine the percentage of apoptosis in cultured cells, significantly reducing the time required for data acquisition and analysis during high throughput experiments. We used this methodology for our high throughput screening to determine possible compounds that would overcome BM-stroma-mediated cytoprotection against daunorubicin.

The Library I-384 from the Merck library of compounds, which includes inhibitors of a variety of pathways involved in cell survival and cell adhesion, was selected for screening. We tested the potential of these compounds to revert the cytoprotection of AML cells mediated by BM stromal cells, as well as their ability to inhibit the proliferation of BM cells. A compound was selected as a positive hit when it fulfilled the following two criteria: (a) It reverted the mCherry-HS5 cells-mediated cytoprotection by increasing the percentage of apoptosis of eGFP–AML cells in co-culture with mCherry-HS5 cells by at least 25% with respect to this heterotypic culture treated with daunorubicin as a single agent; and (b) It inhibited the proliferation of mCherry-HS5 cells by at least 10% with respect to daunorubicin as a single agent (Table 1 and Figure S5). Our data showed that the following pathways from the library under study regulated BM stromal-mediated mechanisms of AML resistance to daurorubicin: PKC kinase, PI3K/Akt, JAK I, CDK1/2/4, and Src kinases, as well as the activity of the nuclear export receptor CRM1/XPO1 (Table 1 and Figure S5).

**Figure 5:**
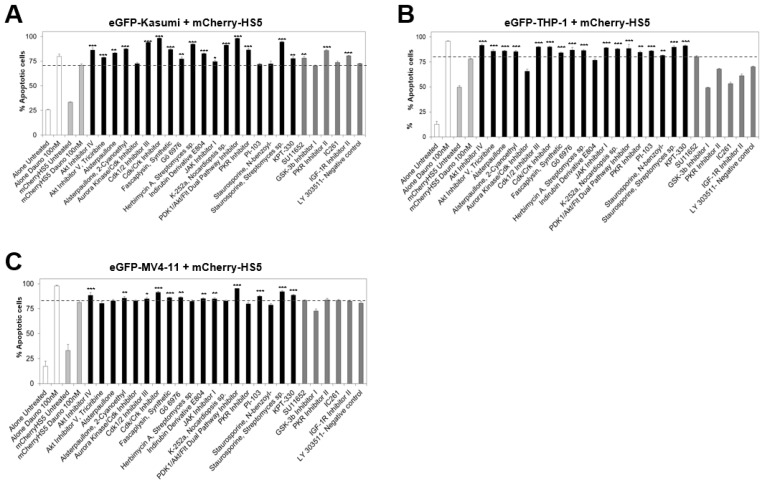
Analysis of the cytotoxic effects of the combination of daunorubicin with the hit compounds identified in the initial drug screening on eGFP–Kasumi, eGFP–THP-1, and eGFP–MV4-11 in co-culture with mCherry-HS5 cells. Graphs show the average percentage and SD of apoptotic eGFP–AML cells cultured alone (white bars) or in the presence of mCherry-HS5 cells (light grey bars) and were left untreated or were treated with 100 nM of daunorubicin. Co-cultures were also treated with 100 nM of daunorubicin in combination with the drugs labelled in the *x* axis. Black bars indicate previously identified hit compounds and dark grey bars, compounds discarded as efficacious in the screening using eGFP–MOLM-14. Graphs show the results using eGFP–Kasumi (**A**), eGFP–THP-1 (**B**), and eGFP–MV4-11 (**C**). * *p* < 0.05, ** *p* < 0.01, *** *p* < 0.005 ANOVA versus eGFP-AML cells in co-culture with mCherry–HS5 treated with 100 nM of daunorubicin.

To identify possible common pathways involved in BM-mediated drug resistance to daunorubicin in AML cells independently of their genetic background, we extended the analysis of the selected hits to a wider panel of eGFP-expressing AML cell lines (eGFP–MV4-11, eGFP–THP-1 and eGFP–Kasumi). As negative controls, we used compounds that were not selected as hits in our initial screening. Eleven compounds (Akt Inhibitor IV, Alsterpaullone, 2-Cyanoethyl, Cdk1/2 Inhibitor III, Cdk/Crk Inhibitor, Fascaplysin, Gö 6976, JAK I, PDK1/Akt/Flt Dual Pathway Inhibitor, the PKC inhibitor Staurosporine Streptomyces sp. and the CRM1 inhibitor KPT-330) showed enhanced killing of daunorubicin-treated AML cells in all the cell lines tested (Figure 5). These inhibitors targeted all the same pathways identified using eGFP-MOLM-14 cells, except for Src and PKR signaling.

**Figure 6:**
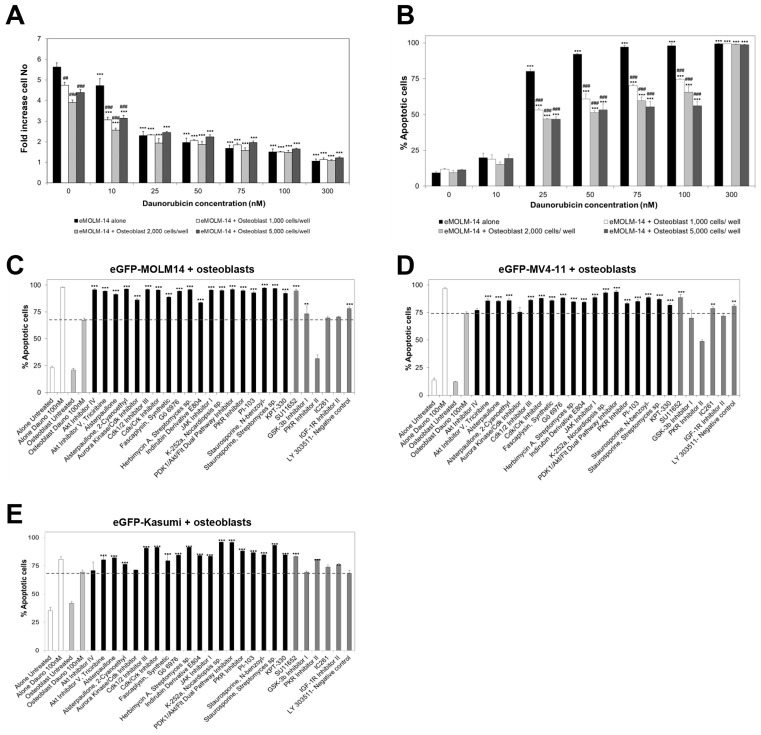
Analysis of the cytotoxic effects of the combination of daunorubicin with the hit compounds identified in the initial drug screening on eGFP–AML cells in co-culture with osteoblasts. The initial optimization of seeding numbers of osteoblasts per well in 96 well plates in co-culture with eGFP–MOLM-14 cells leading to protection against therapeutic drugs, which was tested by measuring the impact of daunorubicin on proliferation (**A**) and the levels of apoptosis (**B**) in eGFP–MOLM-14 cells. In subsequent co-culture experiments, 2000 osteoblasts were seeded per well. A percentage of apoptotic eGFP–MOLM-14 (**C**), eGFP–MV4-11 (**D**), and eGFP–Kasumi (**E**) cells cultured alone (white bars) or in the presence of osteoblasts (light grey bars) when left untreated or when treated with 100 nM daunorubicin. Co-cultures were also treated with 100 nM of daunorubicin in combination with the drugs indicated in the *x* axis. Black bars indicate hit compounds previously identified in our screening using eGFP–MOLM-14, and dark grey bars, compounds discarded as efficacious in that screening. ** *p* < 0.01; *** *p* < 0.005 ANOVA test versus untreated cells under the same culture conditions. ## *p* < 0.01; ### *p* < 0.005 ANOVA test versus the same treatment in monoculture of AML cells.

We then tested the efficacy of the selected hit compounds to overcome the possible cytoprotection of AML cells mediated by osteoblasts. Osteoblasts comprise a critical haematopoietic BM niche that supports the survival of normal haematopoietic stem cells and long-term haematopoietic progenitors, as well as pre-leukaemic stem cells [14]. Therefore, the interaction of AML stem cells with osteoblasts plays a crucial role in AML tumour initiation during the onset of the disease [14] and in resistance to therapeutic drugs [21,22]. Similarly, to the results obtained with mCherry-HS5 cells, the proliferation of AML cells was inhibited by daunorubicin independently of the presence of primary osteoblasts (Figure 6A), while the presence of osteoblasts inhibited the pro-apoptotic effect of daunorubicin (Figure 6B). The combination of daunorubicin with the hit compounds identified using the mCherry-HS5 cells overcame the cytoprotection of all the AML cell lines tested (Figure 6C–E), except for the Akt IV inhibitor.

Finally, we investigated a possible synergistic pro-apoptotic effect of daunorubicin in combination with inhibitors targeting the identified pathways. In the study, we used the inhibitor compounds selected in the high throughput drug screening to overcome cytoprotection mediated by mCherry-HS5 cells and osteoblasts, as well as several clinical inhibitors of the same pathways currently used as therapies or under investigation in clinical trials against various types of cancers. In eGFP–MOLM-14 cells cultured alone, we found some synergistic effect with the combination of daunorubicin and cytarabine (Table 2). However, when these AML cells were treated in heterotypic culture with mCherry-HS5 cells, the synergy between these two drugs was blocked and an antagonistic effect was observed. In contrast, the combination of daunorubicin with the selected hit compounds resulted in a synergistic effect of various degrees that was sustained in the presence of BM cytoprotective cells (Table 2).

Taken together, our data suggest that the inhibition of the pathways regulated by PKC kinase, CDK1 to 7, and CRM1/XPO1 are critical to overcome BM-mediated drug resistance of AML cells against daunorubicin in both the mesenchymal/fibroblastic and osteoblast niches.

### 3.3. In Silico Identification of Potential New KPT-330-Based Combination Therapies against AML

The efficacy of daunorubicin in combination with the CRM1 inhibitor KPT-330 detected in our drug screening has been further validated by recently published preclinical and clinical studies. KPT-330 has been shown to increase in vitro and in vivo the AML-killing efficacy of daunorubicin and other anthracyclins by restoring the nuclear localization of topoisomerase IIα and by downregulating the expression of DNA damage repair genes in AML cells [32]. These results translated into clinical trials, which showed an increased efficacy of the combination of daunorubicin and cytarabine when used with KPT-330 [33,34]. Taken together, these recent in vivo studies and clinical trials strongly support the effectiveness of our high throughput experimental platform and analysis methodology for the identification of efficacious therapies against AML and validate de efficacy of KPT-330 in combination with daunorubicin for treatment of AML patients. However, a recent clinical trial indicates possible limitations in the efficacy of the combination of daunorubicin and KPT-330 for certain AML patient populations [35]. Therefore, new effective KPT-330-based drug combinations may be explored since this CRM1 inhibitor has been shown to be more efficacious in clinical trials when used in combination regimes with additional therapeutic agents [34,36,37].

An overexpression of CRM1 in AML patients inversely correlates with overall survival, constituting a predictive factor for poor prognosis [38]. CRM1 alterations in cancer cells (including mutations and overexpression) are also a common feature associated with poor prognosis in non-haematological tumours [39,40,41,42,43,44,45,46,47,48]. In fact, the compelling preclinical efficacy of KPT-330 in various solid cancer models [49] has led to current clinical trials [47,49]. In order to select drug candidates for screening in combination with KPT-330 in our in vitro AML experimental model, we hypothesized that proteins co-overexpressed with CRM1 in patients with AML, as well as in other tumour types, would represent pro-tumoral pathways co-activated with CRM1 and, therefore, relevant targets for putatively synergistic KPT-330-based combination treatments. We performed an in silico analysis to identify cancer genes whose mRNA levels positively correlate with those of *XPO1,* the gene coding for CRM1 in various solid tumours and AML patients. Specifically, we analyzed the correlation between *XPO1* expression and the expression of a set of 135 cancer-related genes included in the CGA TARGET (Tumour Alterations Relevant for GEnomics-driven Therapy) database of the Broad Institute (https://software.broadinstitute.org/cancer/cga/target (accessed on 19 March 2023)). This database contains genes (hereafter referred to as TARGET genes) with diagnostic, prognostic, or predictive utility whose alteration in cancer is directly linked to a clinical action. Correlation analyses were carried out using publicly available datasets from several cancer types, summarized in Table 3.

**Figure 7:**
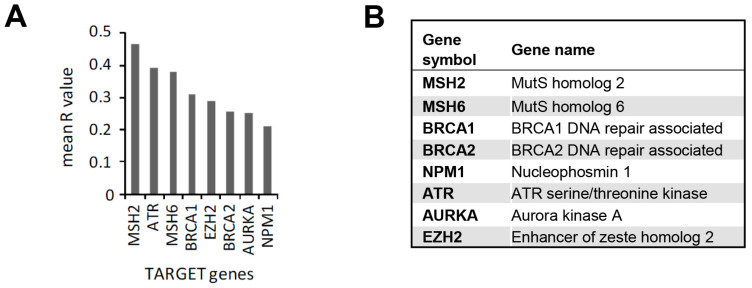
Correlation between the mRNA expression of the *XPO1* gene, coding for CRM1, and the genes in the TARGET dataset. (**A**) Graph showing correlations between the co-expression of the gene coding for CRM1 (*XPO1*) and TARGET genes with a mean R value > 0.2. (**B**) List of the symbol and corresponding complete gene names of identified genes co-expressing with CRM1 with a mean Pearson´s R value > 0.2.

For each type of analyzed tumour type, we selected TARGET genes showing a statistically significant correlation with *XPO1* (*p* ≤ 0.05) with Pearson’s correlation indexes R > 0.2 or R < −0.2 (Supplementary Tables S1–S5). We then calculated the average correlation (mean R value) across the five tumour types (Supplementary Table S6) and identified eight TARGET genes (*MSH2, ATR, MSH6, BRCA1, EZH2, BRCA2, AURKA,* and *NPM1)* whose expression positively correlated with *XPO1* expression in all the tumour types analyzed (Figure 7) that were considered as potential targets in co-treatments with KPT-330. Seven of the identified genes (*MSH2, MSH6, ATR, BRCA1, BRCA2, AURKA,* and *NPM1*) regulate the DNA damage response, while *EZH2* encodes for the protein enhancer of zeste homolog 2 (EZH2), a histone methyltransferase that plays an important role in global transcriptional regulation [69,70]. The overexpression or enhanced activation of EZH2 results in transcriptional repression of tumour suppressor genes [71,72,73,74].

At present, there are no reliable pharmacological inhibitors available to target MSH2, MSH6, BRCA1, BRCA2, or NPM1. On the other hand, there is mounting evidence of AURKA (Aurora Kinase A) and EZH2 proteins as possible targets for cancer therapy in a variety of cancers [75,76,77,78,79,80,81,82] and specific inhibitors, such as alisertib (targeting AURKA) and tazemetostat (targeting EZH2) that are commercially available, which make them ideal compounds for the next steps of drug testing in our system. Importantly, alisertib (MLN8237) is the most clinically advanced AURKA inhibitor currently being tested in clinical trials for cancer treatment [83], and tazemetostat (Tazverik^®^, EPZ-6438, E-7438) has recently received FDA approval for specific subsets of sarcoma and lymphoma patients (https://www.fda.gov/news-events/press-announcements/fda-approves-first-treatment-option-specifically-patients-epithelioid-sarcoma-rare-soft-tissue (accessed on 2 December 2020); https://www.fda.gov/drugs/resources-information-approved-drugs/fda-approves-tazemetostat-advanced-epithelioid-sarcoma (accessed on 2 December 2020); https://www.fda.gov/drugs/fda-granted-accelerated-approval-tazemetostat-follicular-lymphoma (accessed on 2 December 2020).

### 3.4. In Vitro Evaluation of Efficacy of Alisertib and Tazemetostat in Combination with KPT-330-to Overcome BM-Mediated Drug Resistance of AML Cells

**Figure 8:**
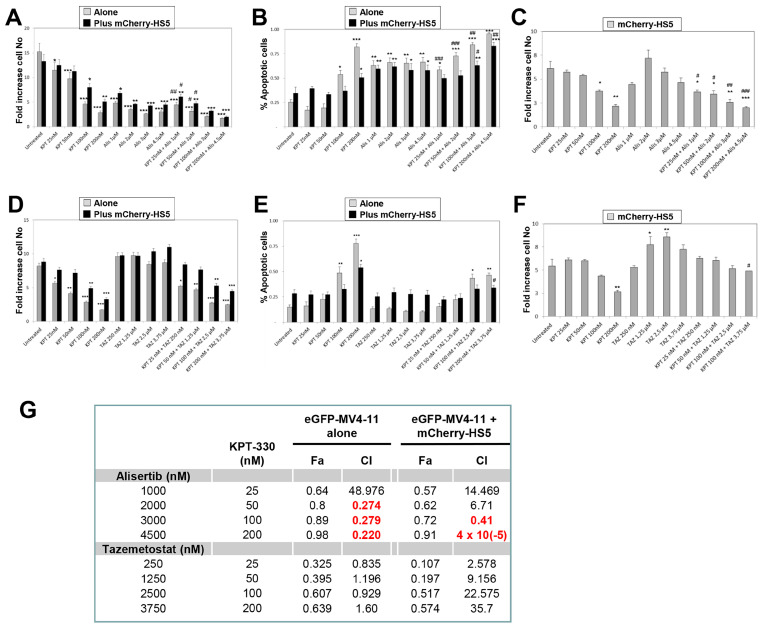
Analysis of the anti-proliferative and pro-apoptotic effect of the combination of KPT-330 with alisertib or tazemetostat. eGFP–MV4-11 cells cultured alone or in the presence of mCherry–HS5 cells were left untreated or were treated with KPT-330 alone or in combination with alisertib (**A**–**C**) or tazemetostat (**D**–**F**). Graphs show the impact of the treatments on the proliferation of eGFP–MV4-11 cells (**A**,**D**), the levels of apoptosis in eGFP–MV4-11 cell cultures (**B**,**E**), and on the proliferation of mCherry–HS5 cells (**C**,**F**). * *p* < 0.05, ** *p* < 0.01, *** *p* < 0.005 ANOVA versus control untreated in the same culture condition. # *p* < 0.05, ## *p* < 0.01, ### *p* < 0.005 post-hoc Student T-test comparing the efficacy of a drug combination with the treatment at the same concentration with KPT-330 as a single agent. (**G**) Table showing the fraction affected (Fa) and combination index (CI) of alisertib or tazemetostat at the specified concentration in combination with 25, 50, 100, and 200 nM of KPT-330. CI values labelled in red indicate synergism or very strong synergism, according to the Chou–Talalay method.

Treatment of eGFP–MV4-11 cells with concentrations of alisertib achievable in patients [84] inhibited their proliferation by at least 75% with minor cytoprotection when cells were cultured in the presence of mCherry-HS5 cells (Figure 8A). The proliferation data correlated with detection of over 70% of cells undergoing apoptosis at all the concentrations tested (Figure 8B), indicating a cytotoxic effect of the treatment. However, alisertib as a single treatment did not affect, or even increase at the lowest concentrations, the proliferation of mCherry-HS5 cells (Figure 8C). In contrast, the combination of KPT-330 with alisertib inhibited the proliferation of mCherry-HS5 cells in a concentration-dependent manner (Figure 8C). This correlated with a more significant decrease in proliferation in comparison with the single treatments (Figure 8A) and a significant increase in the percentage of apoptotic cells in the combination treatment in comparison to the effect of KPT-330 as a single agent when AML cells were cultured alone or in the presence of mCherry-HS5 cells (Figure 8B). A synergistic effect was observed in co-cultured cells at the two higher doses tested (Figure 8G). Of note, treatment with KPT-330 alone blocked the proliferation of mCherry-HS5 cells, which may explain the inhibition of the proliferation of mCherry-HS5 cells when KTP-330 is used in combination with alisertib (Figure 8C) and daunorubicin (Figure S5).

In contrast, when eGFP–MV4-11 were treated with doses of tazemetostat detectable in plasma from patients [85], cells continue to proliferate or even increase their proliferation rate, especially in the presence of mCherry-HS5 cells (Figure 8D). The combination treatment did not enhance the anti-proliferative efficacy of KPT-330 (Figure 8D) or reduce its efficacy to induce apoptosis in eGFP-MV4-11 cells (Figure 8E) or to reduce the proliferation of mCherry-HS5 cells (Figure 8F). This may be due to the pro-proliferative effect of tazemetostat on mCherry-HS5 cells (Figure 8F).

Taken together, our data show increased efficacy of the combination of KPT-330 and alisertib to overcome the cytoprotection by BM stromal cells in comparison to the single agents. However, our results indicate that tazemetostat does not increase the efficacy of KPT-330 and, in fact, the combination with tazemetostat may even exert a detrimental effect on the efficacy of KPT-330.

## 4. Discussion

Numerous studies validate the role of the BM microenvironment in oncogenesis [23,86] and the development of drug resistance in AML [3,12,16,21,22,23,86]. Hence, devising new, improved therapeutic interventions for AML requires the use of significant preclinical in vitro models that recapitulate critical interactions between AML cells and the cytoprotective BM niches. The crosstalk between haematological tumour cells and the local BM stroma leads to reciprocal phenotypic modifications in both cell compartments that favour the expansion of the malignant clones in detriment of normal haematopoiesis [23,86,87]. Therefore, assessing the activity of both cancer and stromal cells using adequate in vitro heterotypic culture models could lead to an improved understanding of the intricate interactions leading to tumour progression and drug resistance.

The BM-derived stromal cell line HS5 largely reproduces critical patterns of activation of BM mesenchymal/fibroblastic stromal cells that contribute to the expansion and survival of malignant clones in different types of haematological malignancies [16,19,20,88]. For example, in the case of MM, the activated transcriptome of MM cells lines in co-culture with HS5 cells, which corresponds to those observed in malignant plasma cells from MM patients [20]. We have previously shown that HS5 cells distinctively activate different cancer cell types that develop tumours in the BM [19,20]. HS5 cells promote the proliferation of MM cells to a much greater extent than other cancer cell types, including chronic myeloid leukaemia (CML), prostate cancer, and breast cancer [19,20]. We now show that HS5 cells do not promote the proliferation of AML cells and even tend to reduce their proliferative potential, similarly to the effect on other myeloid malignancies and in contrast to the impact on MM cells [19]. The use of cancer and stromal cells expressing different fluorescent proteins (eGFP and mCherry, respectively) in our experimental platform allows us to determine the concomitant behaviour of both cellular compartments in co-culture. Using this model presented herein, we found that, similarly to our previous observations with CML cells, AML cells failed to induce the proliferation of HS5 cells. This is in contrast to the pro-proliferative effect of MM cells on HS5 cells [19]. Taken together, our previous and current results validate the reproduction of complex patterns of reciprocal interaction between specific cancer types and stromal cells in the BM niche by heterotypic cultures of tumour cells and HS5 stromal cells.

We then proceeded to use our model for screening of possible compounds that may block the BM stromal-mediated drug resistance of AML cells against daunorubicin, which has been shown to be mimicked by HS5 cells [16]. Our initial findings showed a synergistic effect of daunorubicin and cytarabine to reduce AML proliferation independently of the presence of cytoprotective BM stroma. However, a more detailed analysis of AML cells showed that the lack of proliferation in monocultures of AML cells was due to a pro-apoptotic effect of the drugs, which also synergized for MM cell killing, whereas in the presence of HS5 cells, a significant percentage of the population remained viable albeit non-proliferative. HS5 cells facilitated the cell cycle arrest in G1 and S of AML cells, inhibiting cell cycle progression so that AML cells adopted a non-proliferative phenotype that blocked the pro-apoptotic effect of the drug treatments. In vivo studies have also indicated that the quiescence of AML leukemic stem cells induced by the BM-stroma may be an essential mechanism that promotes resistance to cell proliferation-dependent cytotoxic drugs such as cytarabine [21].

To devise possible strategies to overcome the mechanisms of resistance to daunorubicin, a drug library was screened and compounds were selected by their capacity to block the pro-survival cues of BM stromal cells so that apoptosis was restored to the levels observed in AML cells grown in monoculture, as well as for their ability to reduce the proliferation of HS5 cells in co-culture with AML cells. We have previously shown that a similar strategy works to identify drug combinations to overcome BM-mediated drug resistance in MM [19]. The identified hits were also tested for their capacity to block cytoprotection of AML cells by osteoblasts [23]. The experiments were performed with a battery of eGFP-expressing AML cell lines bearing various representative genetic abnormalities observed in AML patients, as our goal is to identify therapies that could be applied to AML patients independently of their genetic and cytogenetic backgrounds. Our hypothesis is that AML cells may present different strategies based on their genetic background to induce reciprocal interactions with the BM stroma that will lead to the activation of common signaling nodes that promote tumour progression and drug resistance [23].

One such signaling node identified in our screening was the pathway regulated by the CRM1 protein, as its specific inhibitor, KPT-330, was a hit compound in our study. CRM1 is a specialized nuclear export receptor that mediates the translocation of hundreds of cargo proteins bearing nuclear export signals to the cytoplasm (reviewed in [89]). The activity of the CRM1 cargo proteins is regulated by their nucleocytoplasmic distribution and many of them are involved in oncogenesis through the regulation of cell survival, proliferation, and cell adhesion and migration [48,90]. The overexpression of CRM1 is considered a prediction factor for the poor prognosis of AML patients [38], and recent studies show that the combination of KPT-330 with daunorubicin and cytarabine results in increased responses of AML patients to therapy [32,33,34]. These studies further validate the efficacy of our fluorescence-based experimental model and analysis to identify potential improved therapeutic approaches for AML. It has been shown that KPT-330 can enhance the efficacy of daunorubicin and other anthracyclins by interfering with autonomous mechanisms of drug resistance in AML cells involving the DNA repair machinery [32]. Our data suggest that CRM1 may also be involved in the regulation of non-autonomous mechanisms of drug resistance by modulating the interaction of AML cells with cytoprotective BM stromal cells. Possible molecular mechanisms involved in this process may include CRM1 cargo proteins involved in cell adhesion and cytoskeletal remodeling, including N-WASP [91], which has been shown to regulate the adhesive forces of AML cells during the interaction with BM mesenchymal stem cells leading to drug resistance [92].

Importantly, there is recent evidence indicating that the AML therapies based on the combination of KPT-330 with daunorubicin may not be as effective in populations of elderly AML patients [35]. This prompted us to take a combined in silico and in vitro investigation approach to identify new possible alternative combinational therapies based on KPT-330. We then tested in silico the possible co-expression of *XPO1* (gene coding for CRM1) with 135 cancer-related genes included in the CGA TARGET database of the Broad Institute in various types of solid tumours and AML. We reasoned that the co-expression of cancer-related genes and *XPO1* may imply a possible cooperation to promote cancer cell progression and possibly drug resistance. Two of the eight genes identified in this in silico analysis (*AURKA* and *EZH2*) were targets for specific pharmacological inhibitors: the AURKA inhibitor alisertib under testing in clinical trials [83] and the EZH2 inhibitor tazemetostat, which is FDA approved for subsets of sarcoma and lymphoma patients, respectively. Our data showed a synergistic efficacious effect of KPT-330 and alisertib, whereas the combination with tazemetostat failed to increase the anti-AML effect of KPT-330. The efficacy of the KTP-330/alisertib combination has also been recently reported in in vitro and in vivo models of neuroblastoma where these drugs synergize to reactivate p53 activity to induce apoptosis [93]. Further studies should determine whether these or alternative mechanisms of action may be involved in the efficacy of the combination of these two compounds against AML.

## 5. Conclusions

In summary, we have devised a new fluorescence-based experimental model and sequence of procedures to effectively identify new therapeutic approaches for AML. Our method mimics essential reciprocal interactions between AML and cytoprotective BM stromal cells involved in the final response to therapeutic agents. The data obtained suggest that the combination of the CRM1 inhibitor KPT-330 with the AURKA inhibitor alisertib may overcome BM-mediated drug resistance in AML. These data warrant further in vitro and in vivo studies to investigate the possible efficacy of this drug combination against AML.

## Figures and Tables

**Table 1:** Hit compounds that revert BM-mediated drug resistance against daunorubicin of eGFP-MOLM-14 are listed showing the percentage of apoptosis induced on eGFP-MOLM-14 and the percentage of inhibition of mCherry-HS5 cells in co-culture and treated with daunorubicin. (*) Compounds labelled with asterisks partially fulfilled the selection criteria.

	% eGFP-MOLM-14 Apoptotic Cells	% Inhib Prolif mCh-HS5
KPT-330	92.7	57.3
Staurosporine, *Streptomyces* sp. (PKC inhibitor)	99.9	78.1
Staurosporine, N-benzoyl- (PKC inhibitor)	78.1	39.7
PKR Inhibitor	99.9	42.5
PI-103 (PI3K/mTOR inhibitor)	81.4	47.8
PDK1/Akt/Flt Dual Pathway Inhibitor	99.9	87.9
PDGF RTK Inhibitor	76.2	13.5
K-252a, *Nocardiopsis* sp. (PKA, PKC, PKG, others)	100.0	51.2
JAK Inhibitor I	98.1	11.5
Indirubin Derivative E804 (Src-STA3 inhibitor)	80.9	8.9
IKK-2 Inhibitor IV *	81.5	0.8
Herbimycin A, *Streptomyces* sp. (Src inhibitor)	99.2	36.7
GSK-3 Inhibitor XIII *	75.3	0.6
Gö 6976 (PKC inhibitor)	94.9	40.2
Fascaplysin, (CDK4 inhibitor)	82.1	60.1
Cdk1/2 Inhibitor III	99.8	45.2
Cdk/Crk Inhibitor	99.9	56.2
Aurora Kinase/Cdk Inhibitor *	81.9	-15.2
Alsterpaullone, 2-Cyanoethyl (GSK-3β, CDK5/p25, CDK1/cyclin B)	99.7	55
Alsterpaullone * (GSK-3β, CDK5/p25, CDK1/cyclin B)	97.1	6.3
Akt Inhibitor V, Triciribine	81.1	24.7
Akt Inhibitor IV	89.4	56.2

**Table 2:** Analysis of the possible synergistic effect of selected hit compounds. Combination index (CI) and affected fraction (Fa) of the listed compounds are shown at the specified concentration in combination with 10, 25, 50 and 100 nM daunorubicin in eGFP–MOLM-14 cells cultured alone or in co-culture with mCherry-HS5 cells. CI values labelled in red indicate synergism or very strong synergism, according to the Chou–Talalay method. CI values labelled in blue indicate moderate synergism.

		eMOLM-14 Alone		eMOLM-14 + mCherry-HS5	
	Dauno (nM)	Fa	CI	Fa	CI
**Cytarabine**					
100 nM	10	0.709	0.94	0.415	3.083
300 nM	25	0.855	1.446	0.573	2.271
600 nM	50	0.988	**0.754**	0.685	1.79
1 µM	100	0.995	1.005	0.799	1.178
**Cdk/Crk Inhibitor**					
10 nM	10	0.397	1.175	0.452	1.133
20 nM	25	0.877	1.052	0.653	1.104
30 nM	50	0.992	**0.644**	0.828	**0.789**
40 nM	100	0.994	1.082	0.916	**0.601**
**Fascaplysin**					
200 nM	10	0.907	0.925	0.530	1.076
300 nM	25	0.984	0.957	0.766	**0.605**
400 nM	50	0.990	1.337	0.867	**0.537**
600 nM	100	0.993	2.097	0.957	**0.426**
**PDK1/Akt/Flt Inhibitor**					
100 nM	10	0.340	1.522	0.324	2.129
200 nM	25	0.966	**0.834**	0.667	0.988
300 nM	50	0.991	0.917	0.728	1.285
400 nM	100	0.993	1.466	0.975	**0.116**
**Go6976**					
500 nM	10	0.883	**0.605**	0.498	0.753
600 nM	25	0.989	**0.396**	0.795	**0.372**
700 nM	50	0.993	**0.644**	0.837	**0.484**
800 nM	100	0.996	1.031	0.919	**0.39**
**Staurosporine, N-benzoyl**					
200 nM	10	0.975	**0.46**	0.471	0.909
400 nM	25	0.993	**0.441**	0.629	**0.841**
600 nM	50	0.993	**0.791**	0.765	**0.6**
800 nM	100	0.992	1.501	0.916	**0.192**
**Staurosporine, *Streptomyces* sp.**					
5 nM	10	0.992	**0.247**	0.454	1.216
10 nM	25	0.990	**0.84**	0.763	**0.529**
20 nM	50	0.991	1.357	0.901	**0.425**
30 nM	100	0.994	1.279	0.949	**0.363**
**KPT-330**					
100 nM	10	0.756	1.118	0.485	1.097
130 nM	25	0.959	1.024	0.693	**0.748**
160 nM	50	0.992	0.957	0.824	**0.549**
200 nM	100	0.994	1.404	0.929	**0.34**

**Table 3:** List of datasets used in our in silico analysis of the correlation between the expression of *XPO1* and the expression of TARGET genes. Data for solid tumor cohorts are pre-loaded in the CANCERTOOL webtool (http://genomics.cicbiogune.es/CANCERTOOL/citeUs.html, accessed on 11 January 2019). AML data from Liu et al. were retrieved at gdc-portal.nci.nih.gov/legacy-archive/ (accessed on 25 November 2020).

Cancer Type	Study/Reference	Cohort Size	ID
Breast cancer	Lu et al., 2008 [50]	131	GEO: GSE5460
	Ivshina et al., 2016 [51]	249	GEO: GSE4922
	TCGA	522	
	Pawitan et al., 2005 [52]	159	GEO: GSE1456
	Wang et al., 2005 [53]	286	GEO: GSE2034
Lung cancer	Chitale et al., 2009 [54]	128	
	Sheden et al., 2008 [55]	442	GEO: GSE68465
	TCGA	514	
	Wilkerson et al., 2012 [56]	116	GEO: GSE26939
Prostate cancer	Glinsky et al., 2004 [57]	79	
	Grasso et al., 2012 [58]	88	GEO: GSE35988
	Lapointe et al., 2004 [59]	26	GEO: GSE3933
	Taylor et al., 2010 [60]	179	GEO: GSE21034
	TCGA	496	
	Varambally et al., 2005 [61]	19	GEO: GSE3325
Colorectal cancer	Colonomics	246	GEO: GSE44076
	Jorissen et al., 2009 [62]	290	GEO: GSE14333
	Kemper et al., 2012 [63]	90	GEO: GSE33113
	Laibe et al., 2012 [64]	130	GEO: GSE37892
	Marisa et al., 2013 [65]	585	GEO: GSE39582
	Roepman et al., 2014 [66]	188	GEO: GSE42284
	TCGA	374	
Acute myeloid leukaemia (AML)	Tyner et al., 2018 [67]	672	dbGaP: 30641
	Liu et al., 2018 [68]	200	

## Data Availability

The data presented in this study are available in this article and supplementary material.

## Supplementary Materials

The following supporting information can be downloaded at: https://www.mdpi.com/article/10.3390/cancers15071988/s1, Figure S1. Comparison of the sensitivity of estimation of cell numbers by measurement of optical density (OD) in arbitrary units (AU) or of fluorescent intensity emitted by eGFP expressing tumour cells; Figure S2. Expression of eGFP does not affect proliferation rate or drug sensitivity of AML cell lines; Figure S3. Detection of eGFP levels in eGFP-AML cells seeded in the presence of mCherry-HS5 cells; Figure S4. Validation of the measurement of the percentage of low eGFP expressing cells as a readout of the percentage of apoptotic cells in eGFP-AML cell lines; Figure S5. Analysis of the efficacy of compounds in combination with daunorubicin to overcome BM-mesenchymal cell mediated cytoprotection of AML cells; Table S1. Correlation indexes of the co-expression of *XPO1* and TARGET genes (R > 0.2; R < −0.2) with a significant statistically significant value (*p* ≤ 0.05) in analysed breast cancer databases; Supplementary Table S2. Correlation indexes of the co-expression of *XPO1* and TARGET genes (R > 0.2; R < −0.2) with a significant statistically significant value (*p* ≤ 0.05) in analysed lung adenocarcinoma databases; Supplementary Table S3. Correlation indexes of the co-expression of *XPO1* and TARGET genes (R > 0.2; R < −0.2) with a significant statistically significant value (*p* ≤ 0.05) in analysed prostate cancer databases; Supplementary Table S4. Correlation indexes of the co-expression of *XPO1* and TARGET genes (R > 0.2; R < −0.2) with a significant statistically significant value (*p* ≤ 0.05) in analysed colorectal carcinoma primary tumours databases; Supplementary Table S5. Correlation indexes of the co-expression of *XPO1* and TARGET genes (R > 0.2; R < −0.2) with a significant statistically significant value (*p* ≤ 0.05) in analysed AML databases; Supplementary Table S6. Correlation indexes of the co-expression of *XPO1* and TARGET genes (R > 0.2; R < −0.2) with a significant statistically significant value (*p* ≤ 0.05) in all analysed tumour databases.
