# Supplementary material for: High Throughput Fluorescence-Based In Vitro Experimental Platform for the Identification of Effective Therapies to Overcome Tumour Microenvironment-Mediated Drug Resistance in AML
Source: Cancers (Basel). 2023 Mar 27;15(7):1988. doi: 10.3390/cancers15071988 (PMC10093176; doi:10.3390/cancers15071988)
Supplement: Supplementary file 1 [file cancers-15-01988-s001.zip › Supplementary figure and table legends.pdf]

Supplementary figure and table legends

Figure S1. **Comparison of the sensitivity of estimation of cell numbers by measurement of optical density (OD) in arbitrary units (AU) or of fluorescent intensity emitted by eGFP expressing tumour cells.** Correlation between values of OD and cell numbers seeded per well **(A, D, G)**. Correlation between values of fluorescent intensity and numbers of cells seeded per well (**B, E, H**). Correlation between values of OD and fluorescent intensity seeded per well **(C, F, I)**.

Figure S2. **Expression of eGFP does not affect proliferation rate or drug sensitivity of AML cell lines**. Proliferation of parental and eGFP-expressing cells was estimated using MTT assays (OD) or fluorimetry. Histograms show the values of the optical density (OD) in arbitrary units (AU) measured at 570 nm of parental or eGFP-expressing AML cells untreated or treated with daunorubin of MV4-11 and eGFP-MV4-11 (**A, B**), Kasumi and eGFP-Kasumi (**D, E**) and THP-1 and eGFP-THP-1 (**G, H**). Plates used for measurements of optical density were previously used to measure fluorescent intensity of the cultures of eGFP-MV4-11, eGFP-Kasumi and eGFP-THP-1 cells **(C, F, I)**. The results using fluorimetry or spectrometry were equivalent. * $p < 0.05$; ** $p < 0.01$; *** $p < 0.005$ ANOVA test versus control untreated.

Figure S3. **Detection of eGFP levels in eGFP-AML cells seeded in the presence of mCherry-HS5 cells**. Dot plots showing the results of FACS analysis to determine the levels of eGFP and apoptosis (Annexin V positive staining) of eGFP-MV4-11 cells seeded alone **(A, C, E)** or in the presence of mCherry-HS5 cells **(B, D, F)**. Values show the percentage of the total population of cells in each quadrant of the dot plot. Seeding of eGFP-AML cells in heterotypic culture conditions did not alter the levels of eGFP in untreated cells **(A, B)** or when treated with cytotoxic drugs such as daunorubicin (dauno) **(C-F)**.

Figure S4. **Validation of the measurement of the percentage of low eGFP expressing cells as a readout of the percentage of apoptotic cells in eGFP-AML cell lines**. Dot plots show the results of FACS analysis to determine the levels of Annexin V and propidium iodide staining **(A, upper panels),** as well as Annexin V and eGFP **(A, lower panels)** in eGFP-MV4-11 cells seeded alone **(A, C, E)** or in the presence of mCherry-HS5 cells **(B, D, F)**. Values show the percentage of the total population of cells in each quadrant of the dot plot. The eGFP low expressing population in eGFP-AML cells comprises the early (Annexin V positive, propidium iodide weakly stained cells) and late (Annexin V positive, propidium iodide strongly

stained cells) apoptotic cells. **(B)** Graphs showing the direct correlation between the detection of the percentage of eGFP low expressing eGFP-MV4-11 (upper panels) and eGFP-THP-1 (lower panels) and the percentage of Annexin V positive cells. Example of the analysis of the cytotoxic effect of daunorubicin on eGFP-MV4-11 cells using as a readout for the percentage of Annexin V positive cells **(D)** or the percentage of eGFP low expressing cells **(E)**. The results obtained are equivalent using both methodologies. * $p < 0.05$, ** $p < 0.005$, *** $p < 0.001$ ANOVA versus control untreated in the same culture condition. # $p < 0.05$, ## $p < 0.005$, ### $p < 0.001$ post-hoc Student T-test eGFP-MV4-11 cells co-cultured with mCherry-HS5 cells vs eGFP-MV4-11 cells alone under the same dose drug treatment.

Figure S5. **Analysis of the efficacy of compounds in combination with daunorubicin to overcome BM-mesenchymal cell mediated cytoprotection of AML cells. (A)** Analysis of the percentage of apoptotic eGFP-MOLM-14 cells cultured in the presence of mCherry-HS5 and treated with 100 nM daunorubicin (Dauno) alone or in combination with the compounds from the Merck Library I plate. As positive control for the response to daunorubicin, eGFP-MOLM-14 cells were treated with daunorubicin in monoculture (alone); **(B)** Analysis of the proliferation (fold increase in cell numbers in 3 days) of mCherry-HS5 cells cultured in the presence of eGFP-MOLM-14. Efficacious compounds to overcome drug resistance (positive hits) reverted the mCherry-HS5 cells-mediated cytoprotection by increasing the percentage of apoptosis of eGFP-AML cells in co-culture with mCherry-HS5 cells by at least 25% with respect to this heterotypic cultures treated with daunorubicin as a single agent; and inhibited by at least 10% the proliferation of mCherry-HS5 cells treated with daunorubicin as a single agent (bar charts labelled in red). The green labelled bar charts indicate compounds that fully fulfilled only one the criteria. Dashed lines in A show the minimum apoptotic levels for compounds to be considered as hits, and in B the levels of proliferation of mCherry-HS5 cells in co-culture with eGFP-MOLM-14 cells and treated with daunorubicin.

Supplementary Table S1. **Correlation indexes of the co-expression of *XPO1* and TARGET genes ($R > 0.2$; $R < -0.2$) with a significant statistically significant value ($p \leq 0.05$) in analysed breast cancer databases**. The column on the right shows the average correlation value of the databases studied.

Supplementary Table S2. **Correlation indexes of the co-expression of *XPO1* and TARGET genes ($R > 0.2$; $R < -0.2$) with a significant statistically significant value ($p \leq 0.05$) in analysed lung adenocarcinoma databases**. The column on the right shows the average correlation value of the databases studied.

Supplementary Table S3. **Correlation indexes of the co-expression of *XPO1* and TARGET genes (R > 0.2; R < - 0.2) with a significant statistically significant value (p ≤ 0.05) in analysed prostate cancer databases**. The column on the right shows the average correlation value of the databases studied.

Supplementary Table S4. **Correlation indexes of the co-expression of *XPO1* and TARGET genes (R > 0.2; R < - 0.2) with a significant statistically significant value (p ≤ 0.05) in analysed colorectal carcinoma primary tumours databases**. The column on the right shows the average correlation value of the databases studied.

Supplementary Table S5. **Correlation indexes of the co-expression of *XPO1* and TARGET genes (R > 0.2; R < - 0.2) with a significant statistically significant value (p ≤ 0.05) in analysed AML databases**. The column on the right shows the average correlation value of the databases studied.

Supplementary Table S6**. Correlation indexes of the co-expression of *XPO1* and TARGET genes (R > 0.2; R < - 0.2) with a significant statistically significant value (p ≤ 0.05) in all analysed tumour databases**. The column on the right shows the average correlation value of the databases studied.
