# Supplementary material for: High Throughput Fluorescence-Based In Vitro Experimental Platform for the Identification of Effective Therapies to Overcome Tumour Microenvironment-Mediated Drug Resistance in AML
Source: Cancers (Basel). 2023 Mar 27;15(7):1988. doi: 10.3390/cancers15071988 (PMC10093176; doi:10.3390/cancers15071988)
Supplement: Supplementary file 1 [file cancers-15-01988-s001.zip › Supplementary Table S1.pdf]

Supplementary Table S1: Correlation indexes of the co-expression of CRM1 and TARGET genes (R > 0.2; R < - 0.2) with a significant statistically significant value (p ≤ 0.05) in analysed breast cancer databases. The column on the right shows the average correlation value of the databases studied.

	Breast cancer					
	Lu	Ivshina	Pawitan	TCGA	Wang	Average
EZH2	0,288	0,233	0,293	0,473	0,408	0,339
MSH6	0,393	0,222	0,358	0,389	0,283	0,329
AURKA	0,311		0,318	0,417	0,335	0,276
MSH2	0,394		0,275	0,42	0,207	0,259
ATR	0,297		0,257		0,387	0,188
BRCA2	0,308		0,217	0,316		0,168
CCNE1	0,245			0,313	0,249	0,161
BRCA1	0,396			0,366		0,152
RHEB				0,393	0,225	0,124
DNMT3A	0,395				0,205	0,120
NPM1				0,257	0,26	0,103
NRAS				0,225	0,256	0,096
CREBBP					0,313	0,063
BRAF				0,302		0,060
CDK4				0,28		0,056
KRAS					0,274	0,055
GNAS	0,256					0,051
ALK	0,255					0,051
ASXL1			0,248			0,050
SMAD4	0,228					0,046
FLCN			0,226			0,045
EGFR	0,224					0,045
ETV6				0,221		0,044
ROS1	0,221					0,044
PIK3CB					0,218	0,044
IDH2				0,208		0,042
ERRFI1				0,202		0,040
RAB35				0,2		0,040
ATM						0,000
BAP1						0,000
BCL2						0,000
BRD2						0,000
BRD3						0,000
BRD4						0,000
c15orf55						0,000
CCND2						0,000
CCND3						0,000
CDH1						0,000
CDK12						0,000
CDK6						0,000
CDKN1B						0,000

CDKN2A	0,000
CDKN2B	0,000
CRKL	0,000
CTNNB1	0,000
ERBB2	0,000
ERBB4	0,000
ERCC2	0,000
ESR1	0,000
ETV4	0,000
ETV5	0,000
EWSR1	0,000
FBXW7	0,000
FGFR2	0,000
FGFR3	0,000
FLT3	0,000
GNAQ	0,000
HRAS	0,000
IDH1	0,000
IGF1R	0,000
JAK2	0,000
JAK3	0,000
MAP2K1	0,000
MAP2K4	0,000
MAPK1	0,000
MCL1	0,000
MDM2	0,000
MDM4	0,000
	0,000

MED12	
MEN1	0,000
MET	0,000
MLH1	0,000
MLL	0,000
MPL	0,000
MTOR	0,000
MYC	0,000
MYD88	0,000
NF1	0,000
NFKBIA	0,000
NKX2-1	0,000
NOTCH1	0,000
NOTCH2	0,000
NTRK3	0,000
PIK3CA	0,000
PTCH1	0,000
RAF1	0,000
RARA	0,000
RB1	0,000
RET	0,000
RNF43	0,000
RSPO2	0,000
SMAD2	0,000
SMARCA4	0,000
SMARCB1	0,000
SMO	0,000
STK11	0,000
SYK	0,000
TET2	0,000
TMPRSS2	0,000
TP53	0,000

TSC1						0,000
WT1						0,000
ZNRF3						0,000
CEBPA					-0,202	-0,040
ABL1			-0,203			-0,041
ERBB3				-0,205		-0,041
PIK3R1		-0,21				-0,042
AKT2					-0,211	-0,042
NF2	-0,212					-0,042
TSC2					-0,212	-0,042
AR					-0,218	-0,044
KDR				-0,222		-0,044
VHL			-0,224			-0,045
PTEN		-0,23				-0,046
RUNX1				-0,23		-0,046
FGFR1				-0,241		-0,048
ETV1				-0,255		-0,051
MAPK3			-0,259			-0,052
KIT	-0,261					-0,052
EPHA3				-0,27		-0,054
APC				-0,275		-0,055
MAP2K2					-0,278	-0,056
PDGFRA				-0,287		-0,057
AKT3				-0,304		-0,061
MAP3K1			-0,31			-0,062
GNA11	-0,25			-0,247		-0,099
AKT1			-0,282		-0,233	-0,103
ERG	-0,229			-0,289		-0,104
ARAF				-0,228	-0,402	-0,126
CDKN1A	-0,282		-0,434			-0,143
DDR2	-0,271					-0,154

		-0,229		-0,269		
CCND1		-0,258		-0,287		-0,156
			-0,233			
MITF		-0,214	-0,255			-0,172
	-0,19				-0,203	
PDGFRB		-0,257	-0,287		-0,245	-0,216
				-0,289		
