# Supplementary material for: High Throughput Fluorescence-Based In Vitro Experimental Platform for the Identification of Effective Therapies to Overcome Tumour Microenvironment-Mediated Drug Resistance in AML
Source: Cancers (Basel). 2023 Mar 27;15(7):1988. doi: 10.3390/cancers15071988 (PMC10093176; doi:10.3390/cancers15071988)
Supplement: Supplementary file 1 [file cancers-15-01988-s001.zip › Supplementary Table S2.pdf]

Supplementary Table S2: Correlation indexes of the co-expression of CRM1 and TARGET genes (R > 0.2; R < - 0.2) with a significant statistically significant value (p ≤ 0.05) in analysed lung adenocarcinoma cancer databases. The column on the right shows the average correlation value of the databases studied.

	Lung adenocarcinoma				
	Chitale	Shedden	TCGA	Wilkerson	Average
MSH2	0,559	0,715	0,648	0,565	0,622
MSH6	0,564	0,662	0,592	0,58	0,600
ATR	0,508	0,738	0,404	0,476	0,532
EZH2	0,488	0,509	0,516	0,577	0,523
BRCA2	0,484	0,358	0,553	0,555	0,488
AURKA	0,458	0,321	0,482	0,647	0,477
PIK3CA	0,284	0,696	0,33	0,527	0,459
NRAS	0,35	0,491	0,384	0,467	0,423
BRCA1	0,427		0,535	0,651	0,403
CCNE1	0,365		0,427	0,624	0,354
PIK3CB	0,294	0,456		0,384	0,284
BRAF	0,402		0,312	0,413	0,282
CDK12	0,37	0,363	0,361		0,274
IDH1	0,339	0,438		0,247	0,256
DNMT3A	0,328	-0,264	0,392	0,505	0,240
NPM1		0,586		0,215	0,200
					0,198
MAP2K4	0,222	0,514			0,184
ASXL1	0,265	0,202	0,23		0,174
KRAS		0,421	0,264		0,171
CDKN2A	0,415			0,238	0,163
NF1	0,356	0,253			0,152
SMAD4		0,608			0,152
MED12	0,218			0,329	0,137
MAPK1	0,237	0,301			0,135
CRKL				0,528	0,132
MLH1		0,504			0,126
RB1		0,494			0,124
PTEN		0,482			0,121
HRAS	0,225			0,253	0,120
ATM		0,467			0,117

MAP2K2		0,448		0,112
SMAD2		0,432		0,108
SMARCB1			0,398	0,100
CDKN1B		0,388		0,097
FLCN			0,383	0,096
				0,094
ERBB2	0,358			0,090
MEN1	0,349			0,087
AKT1	0,339			0,085

RHEB	0,333				0,083
BRD3	0,298				0,075
CDK4				0,297	0,074
VHL			0,294		0,074
EWSR1	0,29				0,073
IDH2				0,272	0,068
GNAS				0,271	0,068
MAP2K1		0,262			0,066
NOTCH2		0,245			0,061
BRD2	0,241				0,060
RNF43				0,239	0,060
CREBBP	0,234				0,059
CDK6	0,215	-0,516	0,209	0,322	0,058
APC		0,227			0,057
ETV6	0,218				0,055
NF2		-0,272		0,46	0,047
JAK2		0,325		-0,301	0,006
c15orf55					0,000
CDH1					0,000
ERBB3					0,000
ERRFI1					0,000
FGFR3					0,000
GNAQ					0,000
IGF1R					0,000
MAP3K1					0,000
MDM4					0,000
MET					0,000
MLL					0,000
MYC					0,000
NFKBIA					0,000
PTCH1					0,000
RET					0,000
SMARCA4					0,000
TET2					0,000
ZNRF3					0,000
TSC1		0,39		-0,403	-0,003
SMO		-0,396		0,318	-0,020
MTOR	0,212	-0,34			-0,032
CCND1	-0,203				-0,051
TP53				-0,218	-0,055
ETV5			-0,221		-0,055
AKT3	-0,222				-0,056
EPHA3			-0,222		-0,056
TSC2				-0,232	-0,058

NOTCH1		-0,235			-0,059
ETV4		-0,239			-0,060
MDM2		-0,249			-0,062
RAF1	0,388	0,405			
CTNNB1		0,377			
RHEB	0,333				0,083
BRD3	0,298				0,075
CDK4				0,297	0,074
VHL			0,294		0,074
EWSR1	0,29				0,073
IDH2				0,272	0,068
GNAS				0,271	0,068
MAP2K1		0,262			0,066
NOTCH2		0,245			0,061
BRD2	0,241				0,060
RNF43				0,239	0,060
CREBBP	0,234				0,059
CDK6	0,215	-0,516	0,209	0,322	0,058
APC		0,227			0,057
ETV6	0,218				0,055
NF2		-0,272		0,46	0,047
JAK2		0,325		-0,301	0,006
c15orf55					0,000
CDH1					0,000
ERBB3					0,000
ERRFI1					0,000
FGFR3					0,000
GNAQ					0,000
IGF1R					0,000
MAP3K1					0,000
MDM4					0,000
MET					0,000
MLL					0,000
MYC					0,000
NFKBIA					0,000
PTCH1					0,000
RET					0,000
SMARCA4					0,000
TET2					0,000
ZNRF3					0,000
TSC1		0,39		-0,403	-0,003
SMO		-0,396		0,318	-0,020
MTOR	0,212	-0,34			-0,032
CCND1	-0,203				-0,051

TP53				-0,218	-0,055
ETV5			-0,221		-0,055
AKT3	-0,222				-0,056
EPHA3			-0,222		-0,056
TSC2				-0,232	-0,058
NOTCH1		-0,235			-0,059
ETV4		-0,239			-0,060
MDM2		-0,249			-0,062

AKT2		-0,572		0,32	-0,063
BAP1		-0,257			-0,064
ROS1			-0,257		-0,064
FBXW7	-0,264				-0,066
MITF	-0,264				-0,066
EGFR		-0,265			-0,066
PIK3R1	-0,28				-0,070
SYK				-0,284	-0,071
KIT				-0,286	-0,072
FGFR1	-0,299				-0,075
ABL1				-0,322	-0,081
MYD88				-0,34	-0,085
ALK		-0,344			-0,086
MCL1				-0,353	-0,088
BRD4		-0,355			-0,089
WT1		-0,38			-0,095
AR		-0,263		-0,202	-0,116
NKX2-1				-0,473	-0,118
KDR				-0,481	-0,120
ERCC2		-0,489			-0,122
RAB35		-0,492			-0,123
ETV1	-0,28		-0,231		-0,128
ARAF		-0,235	-0,311		-0,137
FGFR2		-0,32	-0,24		-0,140
CDKN2B		-0,363		-0,211	-0,144
PDGFRA	-0,281			-0,301	-0,146
CCND3			-0,268	-0,315	-0,146
TMPRSS2				-0,653	-0,163
RSPO2			-0,279	-0,38	-0,165
DDR2	-0,401	-0,278			-0,170

MPL		-0,681			-0,170
CDKN1A			-0,261	-0,453	-0,179
BCL2	-0,322	-0,403			-0,181
STK11		-0,497	-0,233		-0,183
MAPK3		-0,533	-0,203		-0,184
CEBPA	-0,315		-0,206	-0,253	-0,194
ERBB4	-0,218	-0,3	-0,304		-0,206
ESR1	-0,313	-0,539			-0,213
RUNX1		-0,51		-0,36	-0,218
JAK3	-0,216	-0,672			-0,222
CCND2	-0,276	-0,286	-0,224	-0,211	-0,249
GNA11	-0,312	-0,429		-0,423	-0,291
FLT3	-0,325	-0,632		-0,248	-0,301
PDGFRB	-0,361	-0,319	-0,254	-0,424	-0,340
ERG	-0,398	-0,215	-0,324	-0,496	-0,358
RARA		-0,547	-0,397	-0,5	-0,361
