# Supplementary material for: High Throughput Fluorescence-Based In Vitro Experimental Platform for the Identification of Effective Therapies to Overcome Tumour Microenvironment-Mediated Drug Resistance in AML
Source: Cancers (Basel). 2023 Mar 27;15(7):1988. doi: 10.3390/cancers15071988 (PMC10093176; doi:10.3390/cancers15071988)
Supplement: Supplementary file 1 [file cancers-15-01988-s001.zip › Supplementary Table S3.pdf]

Supplementary Table S3: Correlation indexes of the co-expression of CRM1 and TARGET genes (R > 0.2; R < - 0.2) with a significant statistically significant value (p ≤ 0.05) in analysed prostate cancer databases. The column on the right shows the average correlation value of the databases studied.

	Prostate cancer						
	Glinsky	Grasso	Lapointe	Taylor	TCGA	Varambally	Average
MSH2	0,284	0,414	0,679	0,758	0,623		0,460
RB1		0,474		0,747	0,495	0,824	0,423
FBXW7	0,229			0,635	0,426	0,922	0,369
ATM				0,796	0,571	0,795	0,360
CTNNB1			0,834	0,858	0,314		0,334
ATR		0,366		0,825	0,592		0,297
NOTCH2			0,573	0,572	0,49		0,273
DDR2				0,373	0,338	0,805	0,253
MDM4		-0,312	0,659	0,717	0,44		0,251
PIK3CA				0,837	0,569		0,234
NF1				0,782	0,546		0,221
BRAF				0,759	0,553		0,219
TET2				0,622	0,656		0,213
MLH1		0,499		0,766			0,211
MSH6				0,725	0,533		0,210
PDGFRA				0,279		0,973	0,209
KRAS				0,706	0,534		0,207
GNAQ				0,827	0,371		0,200
APC				0,779	0,403		0,197
CCND2				0,252	0,926		0,196
SMAD2				0,77	0,369		0,190
MAP3K1				0,604	0,527		0,189
NRAS				0,724	0,347		0,179
MAPK1				0,69	0,374		0,177
VHL				0,518	0,533		0,175
PIK3CB				0,742	0,287		0,172
CDKN1B	0,355			0,411	0,262		0,171
JAK2				0,62	0,374		0,166
RAF1				0,719	0,247		0,161
CDK12				0,47	0,487		0,160
PIK3R1				0,661	0,262		0,154
MDM2				0,562	0,335		0,150
MYC			0,66	0,225			0,148
BRD2				0,423	0,435		0,143
CREBBP				0,365	0,493		0,143
MCL1				0,598	0,216		0,136
PDGFRB						0,797	0,133
NPM1				0,735			0,123
RHEB				0,707			0,118
MAP2K4	-0,239	0,284		0,648			0,116
AKT3				0,682			0,114

SMAD4			0,682			0,114
CRKL			0,204	0,448		0,109
PTEN			0,62			0,103
IDH1			0,578			0,096
PTCH1				0,533		0,089
BRCA1				0,527		0,088
EZH2	0,243	0,658		0,288	-0,684	0,084
CDH1			0,497			0,083
MED12				0,415		0,069
IDH2				-0,397	0,806	0,068
ESR1			-0,411		0,809	0,066
EPHA3			0,394			0,066
EWSR1			0,394			0,066
MAP2K1			0,389			0,065
GNAS			0,378			0,063
ASXL1				0,365		0,061
KDR			0,323			0,054
BRCA2			-0,294	0,612		0,053
KIT			0,31			0,052
MET			0,31			0,052
GNA11				0,292		0,049
ERRFI1			0,291			0,049
ETV6			0,274			0,046
CDK4			0,267			0,045
TSC2	0,267					0,045
MYD88				0,249		0,042
SYK				0,237		0,040
DNMT3A	0,243		-0,426	0,418		0,039
MITF			0,211			0,035
NOTCH1				0,209		0,035
ABL1	-0,308		0,221	0,282		0,033
TSC1		-0,596	0,358	0,345		0,018
AR			0,309	0,521	-0,791	0,007

NF2		-0,301	0,306			0,001
BCL2						0,000
c15orf55						0,000
ETV1						0,000
ETV5						0,000
FGFR2						0,000
FLCN	0,273		-0,645	0,372		0,000
MLL						0,000
MPL						0,000
RAB35						0,000
SMARCA4						0,000
TP53						0,000
BRD4			-0,311	0,303		-0,001
AURKA	0,257		0,221	0,299	-0,872	-0,016
BRD3		-0,301	-0,335	0,505		-0,022
FLT3		0,299	-0,455			-0,026
NTRK3			-0,22			-0,037
NFKBIA	-0,231					-0,039
RSPO2				-0,239		-0,040
ERG			0,232	0,205	-0,689	-0,042
RET			-0,274			-0,046
MAPK3				-0,29		-0,048
RUNX1			-0,29			-0,048
EGFR			0,232	0,396	-0,925	-0,050
MTOR		-0,298				-0,050
AKT1	-0,301					-0,050
ROS1			-0,622	0,316		-0,051
ERBB4			-0,322			-0,054
CCND1		-0,335				-0,056
ZNRF3		-0,384				-0,064
RNF43		-0,405				-0,068
ARAF				-0,507		-0,085
IGF1R			0,282		-0,792	-0,085
NKX2-1			-0,759			-0,091

					0,214		
MEN1				-0,55			-0,092
ETV4				-0,332			-0,095
					-0,236		
WT1				-0,593			-0,099
CDKN2A				-0,599			-0,100
FGFR3				-0,626			-0,104
CCNE1				-0,631			-0,105
BAP1				-0,428			-0,107
					-0,211		
ALK				-0,667			-0,111
AKT2				-0,369			-0,119
		-0,346					
CDKN2B				-0,735			-0,123
TMPRSS2							-0,125
			-0,751				
STK11							-0,127
				-0,414	-0,349		
ERCC2				-0,563	-0,242		-0,134
CDK6				0,283			-0,135
		-0,474	-0,62				
ERBB2			-0,601				-0,139
	-0,233						
FGFR1	-0,244		-0,605				-0,142
RARA	-0,269						-0,152
				-0,641			
JAK3				-0,418			-0,179
			-0,656				
CEBPA				-0,617			-0,195
	0,227	-0,373			-0,408		
ERBB3		-0,325					-0,198
						-0,865	
CDKN1A							-0,207
	-0,373		-0,604		-0,264		
SMO	-0,278						-0,230
		-0,477		-0,622			
SMARCB1				-0,256			-0,260
					-0,378	-0,928	
HRAS				-0,717	-0,497		-0,270
		-0,406					
MAP2K2				-0,328	-0,627		-0,288
						-0,771	
CCND3				-0,603	-0,303	-0,843	-0,292
