# Supplementary material for: High Throughput Fluorescence-Based In Vitro Experimental Platform for the Identification of Effective Therapies to Overcome Tumour Microenvironment-Mediated Drug Resistance in AML
Source: Cancers (Basel). 2023 Mar 27;15(7):1988. doi: 10.3390/cancers15071988 (PMC10093176; doi:10.3390/cancers15071988)
Supplement: Supplementary file 1 [file cancers-15-01988-s001.zip › Supplementary Table S4.pdf]

Supplementary Table S4: Correlation indexes of the co-expression of CRM1 and TARGET genes (R > 0.2; R < - 0.2) with a significant statistically significant value (p ≤ 0.05) in analysed colorectal carcinoma primary tumours databases. The column on the right shows the average correlation value of the databases studied.

Colorectal Primary Tumours

	Colon-omics	Jorissen	Kemper	Laibe	Marisa	Roepman	TCGA	Average
ATR	0,387	0,201	0,404	0,369	0,572	0,354	0,389	0,382
BRCA1	0,554		0,275	0,405	0,452	0,388	0,521	0,371
EZH2	0,458		0,325	0,481	0,468	0,373	0,389	0,356
MSH2	0,573		0,276	0,211	0,433	0,466	0,509	0,353
BRCA2	0,599		0,261	0,445	0,528		0,5	0,333
NPM1	0,486		0,312	0,269	0,35	0,215	0,27	0,272
AURKA	0,448			0,373	0,349	0,296	0,433	0,271
MSH6	0,592				0,356	0,277	0,521	0,249
CDK12	0,429	0,286		0,289	0,385		0,331	0,246
BRAF		0,201		0,366	0,518		0,613	0,243
RAF1		0,301	0,427	0,501	0,405			0,233
ASXL1		0,337	0,352	0,507	0,429			0,232
CTNNB1	0,4	0,228		0,214	0,231	0,345		0,203
CCNE1	0,294	0,209		0,324		0,257	0,33	0,202
EWSR1	0,4			0,437	0,221	0,25		0,187
MYC	0,496			0,333			0,26	0,156
ATM			0,245	0,227	0,272		0,247	0,142
CDK4	0,292			0,278			0,278	0,121
CDKN1B	0,273			0,276	0,296			0,121
FBXW7				0,253	0,365	0,224		0,120
TSC1		0,252		0,283	0,306			0,120
NRAS	0,267				0,251	0,297		0,116
NF1	0,235			0,273			0,286	0,113
GNAS			0,234	0,272	0,273			0,111
PIK3CB	0,259				0,306	0,212		0,111
RHEB			0,298		0,226	0,242		0,109
SMARCA4	0,399			0,347				0,107
MDM4				0,361	0,341			0,100
RB1	0,329				0,305			0,091
VHL	0,288						0,312	0,086
RNF43	0,206			0,325				0,076
IDH1	0,261					0,211		0,067
BRD4	0,268			0,2				0,067
MTOR	0,216		0,238					0,065
HRAS	0,202					0,239		0,063
PIK3CA					0,368			0,053
MET	0,36							0,051
MLH1	0,346							0,049
NOTCH1				0,321				0,046
ETV4				0,281				0,040
CDK6					0,277			0,040

KRAS					0,271			0,039
MED12				0,251				0,036
PTCH1	-0,221			0,268	0,202			0,036
PTEN					0,247			0,035
AKT1						0,246		0,035
CDKN2A						0,238		0,034
ETV1						0,238		0,034
SYK				0,273	0,206	-0,241		0,034
CCND1	0,235							0,034
FLCN				0,226				0,032
BRD2							0,22	0,031
JAK2					0,207			0,030
SMAD4					0,206			0,029
BRD3		0,261		0,257		-0,341		0,025
MAP2K1			-0,216			0,374		0,023
MEN1	0,347					-0,236		0,016
AKT2								0,000
ARAF								0,000
BAP1								0,000
c15orf55								0,000
CCND2								0,000
CCND3								0,000
CDH1								0,000
CEBPA								0,000
CREBBP								0,000
DNMT3A								0,000
EGFR								0,000
ERBB3								0,000
ERRFI1								0,000
ETV5								0,000
FGFR3								0,000
IGF1R								0,000
PIK3R1								0,000
SMO								0,000
TMPRSS2								0,000
TP53								0,000
WT1								0,000
ZNRF3								0,000
ABL1								-0,012

		0,248	-0,329					
MAPK1	0,3		-0,264	-0,21				-0,025
MDM2			-0,219					-0,031
RET	-0,231							-0,033
NKX2-1					-0,236			-0,034
IDH2			-0,243					-0,035
ROS1					-0,243			-0,035
FLT3	-0,247							-0,035
ERBB2		-0,249						-0,036
ERCC2					-0,249			-0,036
NOTCH2			-0,249					-0,036
STK11					-0,249			-0,036
CRKL			-0,252					-0,036
NF2					-0,271			-0,039
APC				-0,277				-0,040
ERBB4					-0,292			-0,042
MLL						-0,319		-0,046
PDGFRB	-0,328							-0,047
SMAD2		-0,334		-0,236		0,241		-0,047
AKT3	-0,336							-0,048
KDR			-0,351					-0,050
ERG	-0,395							-0,056
SMARCB1					-0,424			-0,061
NFKBIA			-0,216		-0,216			-0,062
ETV6						-0,215	-0,236	-0,064
MAP3K1	-0,222			-0,234				-0,065
RUNX1	-0,222					-0,252		-0,068
TET2								-0,068

		-0,213		-0,262				
ALK	-0,212	-0,294						-0,072
MYD88				-0,248<br>-0,261	-0,263			-0,073
PDGFRA	-0,261							-0,075
KIT			-0,263	-0,266				-0,076
MAP2K4			-0,243	-0,292				-0,076
RAB35		-0,245			-0,296			-0,077
MCL1			-0,217	-0,345<br>-0,251				-0,080
EPHA3	-0,314							-0,081
FGFR1	-0,37					-0,238		-0,087
MITF	-0,357			-0,251				-0,087
RSPO2	-0,212			-0,2	-0,208			-0,089
MPL			-0,243		-0,403			-0,092
ESR1					-0,41	-0,279		-0,098
AR			-0,215	-0,263	-0,232			-0,101
GNA11			-0,219	-0,206	-0,291			-0,102
GNAQ			-0,424	-0,296				-0,103
JAK3	-0,221				-0,287	-0,308		-0,117
DDR2	-0,362			-0,212		-0,25		-0,118
TSC2	-0,234					-0,378	-0,272	-0,126
NTRK3	-0,2	-0,206			-0,389	-0,208		-0,143
MAP2K2			-0,223		-0,351			-0,162
RARA	-0,242		-0,221		-0,359	-0,374		-0,171
CDKN1A			-0,384	-0,32<br>-0,382	-0,273		-0,251	-0,175
BCL2	-0,221	-0,207	-0,297		-0,209			-0,188
FGFR2	-0,466		-0,253	-0,31<br>-0,252	-0,332	-0,29		-0,236
MAPK3	-0,413	-0,253			-0,385		-0,421	-0,246
CDKN2B	-0,436			-0,421<br>-0,269			-0,222<br>-0,29	-0,154
