# Supplementary material for: High Throughput Fluorescence-Based In Vitro Experimental Platform for the Identification of Effective Therapies to Overcome Tumour Microenvironment-Mediated Drug Resistance in AML
Source: Cancers (Basel). 2023 Mar 27;15(7):1988. doi: 10.3390/cancers15071988 (PMC10093176; doi:10.3390/cancers15071988)
Supplement: Supplementary file 1 [file cancers-15-01988-s001.zip › Supplementary Table S5.pdf]

Supplementary Table S5: Correlation indexes of the co-expression of CRM1 and TARGET genes (R > 0.2; R < - 0.2) with a significant statistically significant value (p ≤ 0.05) in analysed AML databases. The column on the right shows the average correlation value of the databases studied.

	AML		
	Liu	Tyner	Average
MSH2	0,624	0,661	0,642
ATR	0,557	0,552	0,554
BRCA1	0,583	0,489	0,536
CDK6	0,505	0,531	0,518
MSH6	0,581	0,429	0,505
MLH1	0,393	0,484	0,439
NF1	0,509	0,209	0,359
NPM1	0,403	0,304	0,354
TP53		0,693	0,347
RUNX1	0,286	0,387	0,337
MEN1		0,658	0,329
FLT3	0,226	0,431	0,328
BCL2	0,291	0,351	0,321
RB1	0,380	0,246	0,313
SMARCA4		0,595	0,297
IDH1		0,580	0,290
CDK4		0,572	0,286
SMAD4	0,570		0,285
ERCC2		0,566	0,283
MTOR		0,559	0,280
PTEN	0,350	0,206	0,278
BRD3		0,533	0,266
AURKA	0,221	0,298	0,259
GNAQ	0,509		0,255
KIT	0,279	0,230	0,254
BRCA2	0,484		0,242
MED12		0,451	0,226
MYC		0,439	0,219
SMAD2	0,416		0,208
IDH2		0,380	0,190
ERBB2		0,375	0,188
MAP2K4	0,339		0,170
ZNRF3		0,332	0,166
DNMT3A		0,311	0,155
EZH2	0,303		0,151
SMARCB1		0,297	0,148
PIK3CB	0,271		0,136
CRKL	0,271		0,135
PIK3R1	0,510	-0,271	0,119
FGFR3		0,234	0,117

MDM4	0,221		0,111
CEBPA		0,221	0,110
ETV6		0,209	0,104
ATM		0,202	0,101
EWSR1		0,201	0,100
NRAS	0,47 8	-0,339	0,070
CDK12	0,371	-0,286	0,043
GNAS	-0,240	0,250	0,005
APC	0,331	-0,328	0,001
ALK			0,000
AR			0,000
ASXL1			0,000
c15orf55			0,000
CCND1			0,000
CCND2			0,000
CCNE1			0,000
CDH1			0,000
CDKN2A			0,000
CDKN2B			0,000
DDR2			0,000
EGFR			0,000
EPHA3			0,000
ERBB3			0,000
ERBB4			0,000
ERG			0,000
ESR1			0,000
ETV1			0,000
ETV4			0,000
ETV5			0,000
FGFR2			0,000
IGF1R			0,000
JAK2			0,000
KDR			0,000
MAP3K1			0,000
MAPK1			0,000
MET			0,000
MITF			0,000
MLL			0,000
MPL			0,000
NF2			0,000
NKX2-1			0,000
NOTCH2			0,000
NTRK3			0,000
PDGFRA			0,000
PDGFRB			0,000
RNF43			0,000

ROS1			0,000
RSPO2			0,000
SMO			0,000
SYK			0,000
TMPRSS2			0,000
TSC1			0,000
WT1			0,000
TSC2	-0,334	0,328	-0,003
STK11	-0,286	0,254	-0,016
MDM2	0,202	-0,242	-0,020
BRAF	0,392	-0,486	-0,047
AKT1	-0,448	0,325	-0,062
PIK3CA	0,396	-0,549	-0,077
KRAS	0,390	-0,568	-0,089
FGFR1		-0,204	-0,102
PTCH1	-0,206		-0,103
NOTCH1	-0,219		-0,110
RET	-0,231		-0,116
TET2		-0,237	-0,119
AKT3		-0,239	-0,120
CDKN1B		-0,263	-0,131
GNA11	-0,269		-0,135
RHEB		-0,270	-0,135
CTNNB1		-0,277	-0,139
JAK3	-0,302		-0,151
BRD4	-0,304		-0,152
BRD2	-0,331		-0,165
ERRFI1		-0,339	-0,170
FLCN	-0,359		-0,180
AKT2	-0,397		-0,198
VHL		-0,400	-0,200
CCND3	-0,418		-0,209
MCL1		-0,438	-0,219
HRAS	-0,479		-0,240
FBXW7		-0,572	-0,286
BAP1	-0,361	-0,259	-0,310
MAP2K1	-0,311	-0,334	-0,323
MYD88	-0,412	-0,236	-0,324
MAP2K2	-0,468	-0,216	-0,342
CDKN1A	-0,411	-0,346	-0,379
CREBBP	-0,221	-0,587	-0,404
RAB35	-0,471	-0,341	-0,406
ARAF	-0,556	-0,309	-0,432
NFKBIA	-0,419	-0,491	-0,455
RAF1	-0,512	-0,476	-0,494

MAPK3	-0,597	-0,461	-0,529
