# Supplementary material for: High Throughput Fluorescence-Based In Vitro Experimental Platform for the Identification of Effective Therapies to Overcome Tumour Microenvironment-Mediated Drug Resistance in AML
Source: Cancers (Basel). 2023 Mar 27;15(7):1988. doi: 10.3390/cancers15071988 (PMC10093176; doi:10.3390/cancers15071988)
Supplement: Supplementary file 1 [file cancers-15-01988-s001.zip › Supplementary Table S6.pdf]

Supplementary Table S6: Correlation indexes of the co-expression of CRM1 and TARGET genes (R > 0.2; R < - 0.2) with a significant statistically significant value (p ≤ 0.05) in all analysed tumour databases. The column on the right shows the average correlation value of the databases studied.

	All tumours					
	**Breast**	**Lung**	**Prostate**	**Colorectal**	**AML**	**Average**
MSH2	0,259	0,622	0,460	0,353	0,642	0,467
ATR	0,188	0,532	0,297	0,382	0,554	0,391
MSH6	0,329	0,600	0,210	0,249	0,505	0,379
BRCA1	0,152	0,403	0,088	0,371	0,536	0,310
EZH2	0,339	0,523	0,084	0,356	0,151	0,291
BRCA2	0,168	0,488	0,053	0,333	0,242	0,257
AURKA	0,276	0,477	-0,016	0,271	0,259	0,254
NPM1	0,103	0,200	0,123	0,272	0,354	0,210
RB1	0,000	0,124	0,423	0,091	0,313	0,190
NRAS	0,096	0,423	0,179	0,116	0,070	0,177
NF1	0,000	0,152	0,221	0,113	0,359	0,169
MLH1	0,000	0,126	0,211	0,049	0,439	0,165
BRAF	0,060	0,282	0,219	0,243	-0,047	0,151
PIK3CB	0,044	0,284	0,172	0,111	0,136	0,149
CDK12	0,000	0,274	0,160	0,246	0,043	0,144
ATM	0,000	0,117	0,360	0,142	0,101	0,144
IDH1	0,000	0,256	0,096	0,067	0,290	0,142
PIK3CA	0,000	0,459	0,234	0,053	-0,077	0,134
SMAD4	0,046	0,152	0,114	0,029	0,285	0,125
CCNE1	0,161	0,354	-0,105	0,202	0,000	0,122
CDK4	0,056	0,074	0,045	0,121	0,286	0,116
DNMT3A	0,120	0,240	0,039	0,000	0,155	0,111
MYC	0,000	0,000	0,148	0,156	0,219	0,104
ASXL1	0,050	0,174	0,061	0,232	0,000	0,103
CTNNB1	0,000	0,094	0,334	0,203	-0,139	0,098
PTEN	-0,046	0,121	0,103	0,035	0,278	0,098
CDK6	0,000	0,058	-0,135	0,040	0,518	0,096
MED12	0,000	0,137	0,069	0,036	0,226	0,093
MDM4	0,000	0,000	0,251	0,100	0,111	0,092
SMAD2	0,000	0,108	0,190	-0,047	0,208	0,092
EWSR1	0,000	0,073	0,066	0,187	0,100	0,085
SMARCA4	0,000	0,000	0,000	0,107	0,297	0,081
MAP2K4	0,000	0,184	0,116	-0,076	0,170	0,079
KRAS	0,055	0,171	0,207	0,039	-0,089	0,077
GNAQ	0,000	0,000	0,200	-0,103	0,255	0,070
BRD3	0,000	0,075	-0,022	0,025	0,266	0,069
MEN1	0,000	0,087	-0,092	0,016	0,329	0,068
CRKL	0,000	0,132	0,109	-0,036	0,135	0,068
IDH2	0,042	0,068	0,068	-0,035	0,190	0,067
RHEB	0,124	0,083	0,118	0,109	-0,135	0,060

NOTCH2	0,000	0,061	0,273	-0,036	0,000	0,060
GNAS	0,051	0,068	0,063	0,111	0,005	0,060
TP53	0,000	-0,055	0,000	0,000	0,347	0,058
MAPK1	0,000	0,135	0,177	-0,025	0,000	0,057
MTOR	0,000	-0,032	-0,050	0,065	0,280	0,053
CDKN1B	0,000	0,097	0,171	0,121	-0,131	0,052
JAK2	0,000	0,006	0,166	0,030	0,000	0,040
ETV6	0,044	0,055	0,046	-0,064	0,104	0,037
PIK3R1	-0,042	-0,070	0,154	0,000	0,119	0,032
APC	-0,055	0,057	0,197	-0,040	0,001	0,032
FBXW7	0,000	-0,066	0,369	0,120	-0,286	0,027
TSC1	0,000	-0,003	0,018	0,120	0,000	0,027
KIT	-0,052	-0,072	0,052	-0,076	0,254	0,021
MET	0,000	0,000	0,052	0,051	0,000	0,021
ERBB2	0,000	0,090	-0,139	-0,036	0,188	0,021
ZNRF3	0,000	0,000	-0,064	0,000	0,166	0,020
RAF1	0,000	0,198	0,161	0,233	-0,494	0,020
CDKN2A	0,000	0,163	-0,100	0,034	0,000	0,019
VHL	-0,045	0,074	0,175	0,086	-0,200	0,018
CDH1	0,000	0,000	0,083	0,000	0,000	0,017
BRD2	0,000	0,060	0,143	0,031	-0,165	0,014
RNF43	0,000	0,060	-0,068	0,076	0,000	0,014
MAP3K1	-0,062	0,000	0,189	-0,065	0,000	0,012
MDM2	0,000	-0,062	0,150	-0,031	-0,020	0,007
TET2	0,000	0,000	0,213	-0,068	-0,119	0,005
PTCH1	0,000	0,000	0,089	0,036	-0,103	0,004
FGFR3	0,000	0,000	-0,104	0,000	0,117	0,003
SYK	0,000	-0,071	0,040	0,034	0,000	0,001
c15orf55	0,000	0,000	0,000	0,000	0,000	0,000
FLCN	0,045	0,096	0,000	0,032	-0,180	-0,001
ERCC2	0,000	-0,122	-0,134	-0,036	0,283	-0,002
NF2	-0,042	0,047	0,001	-0,039	0,000	-0,007
FLT3	0,000	-0,301	-0,026	-0,035	0,328	-0,007
RUNX1	-0,046	-0,218	-0,048	-0,068	0,337	-0,009
MLL	0,000	0,000	0,000	-0,046	0,000	-0,009
BCL2	0,000	-0,181	0,000	-0,188	0,321	-0,010
CCND2	0,000	-0,249	0,196	0,000	0,000	-0,011
ETV5	0,000	-0,055	0,000	0,000	0,000	-0,011
PDGFRA	-0,057	-0,146	0,209	-0,075	0,000	-0,014
EGFR	0,045	-0,066	-0,050	0,000	0,000	-0,014
SMARCB1	0,000	0,100	-0,260	-0,061	0,148	-0,015
ERRFI1	0,040	0,000	0,049	0,000	-0,170	-0,016
IGF1R	0,000	0,000	-0,085	0,000	0,000	-0,017
NOTCH1	0,000	-0,059	0,035	0,046	-0,110	-0,018
AKT1	-0,103	0,085	-0,050	0,035	-0,062	-0,019
					0,000	-0,020

ETV4	0,000	-0,060	-0,095	0,040	0,000	-0,023
EPHA3	-0,054	-0,056	0,066	-0,081	0,000	-0,025
CREBBP	0,063	0,059	0,143	0,000	-0,404	-0,028
ETV1	-0,051	-0,128	0,000	0,034	0,000	-0,029
KDR	-0,044	-0,120	0,054	-0,050	0,000	-0,032
MAP2K1	0,000	0,066	0,065	0,023	-0,323	-0,034
AKT3	-0,061	-0,056	0,114	-0,048	-0,120	-0,034
BRD4	0,000	-0,089	-0,001	0,067	-0,152	-0,035
TSC2	-0,042	-0,058	0,045	-0,126	-0,003	-0,037
DDR2	-0,154	-0,170	0,253	-0,118	0,000	-0,038
WT1	0,000	-0,095	-0,099	0,000	0,000	-0,039
RET	0,000	0,000	-0,046	-0,033	-0,116	-0,039
ALK	0,051	-0,086	-0,111	-0,072	0,000	-0,044
CCND1	-0,156	-0,051	-0,056	0,034	0,000	-0,046
ERBB3	-0,041	0,000	-0,198	0,000	0,000	-0,048
NKX2-1	0,000	-0,118	-0,091	-0,034	0,000	-0,049
ESR1	0,000	-0,213	0,066	-0,098	0,000	-0,049
SMO	0,000	-0,020	-0,230	0,000	0,000	-0,050
MCL1	0,000	-0,088	0,136	-0,080	-0,219	-0,050
AR	-0,044	-0,116	0,007	-0,101	0,000	-0,051
MPL	0,000	-0,170	0,000	-0,092	0,000	-0,053
TMPRSS2	0,000	-0,163	-0,125	0,000	0,000	-0,058
MITF	-0,172	-0,066	0,035	-0,087	0,000	-0,058
RSPO2	0,000	-0,165	-0,040	-0,089	0,000	-0,059
ERBB4	0,000	-0,206	-0,054	-0,042	0,000	-0,060
CEBPA	-0,040	-0,194	-0,195	0,000	0,110	-0,064
HRAS	0,000	0,120	-0,270	0,063	-0,240	-0,065
STK11	0,000	-0,183	-0,127	-0,036	-0,016	-0,072
FGFR2	0,000	-0,140	0,000	-0,236	0,000	-0,075
CDKN2B	0,000	-0,144	-0,123	-0,154	0,000	-0,084
AKT2	-0,042	-0,063	-0,119	0,000	-0,198	-0,085
MYD88	0,000	-0,085	0,042	-0,073	-0,324	-0,088
FGFR1	-0,048	-0,075	-0,142	-0,087	-0,102	-0,091
PDGFRB	-0,216	-0,340	0,133	-0,047	0,000	-0,094
BAP1	0,000	-0,064	-0,107	0,000	-0,310	-0,096
NFKBIA	0,000	0,000	-0,039	-0,062	-0,455	-0,111
ERG	-0,104	-0,358	-0,042	-0,056	0,000	-0,112
RAB35	0,040	-0,123	0,000	-0,077	-0,406	-0,113
GNA11	-0,099	-0,291	0,049	-0,102	-0,135	-0,116
NTRK3	0,000	-0,434	-0,037	-0,143	0,000	-0,123
CCND3	0,000	-0,146	-0,292	0,000	-0,209	-0,129
JAK3	0,000	-0,222	-0,179	-0,117	-0,151	-0,134
MAP2K2	-0,056	0,112	-0,288	-0,162	-0,342	-0,147
ARAF	-0,126	-0,137	-0,085	0,000	-0,432	-0,156
MAPK3	-0,052	-0,184	-0,048	-0,246	-0,529	-0,212
					-0,379	-0,217
