# Supplementary figures and images for: High Throughput Fluorescence-Based In Vitro Experimental Platform for the Identification of Effective Therapies to Overcome Tumour Microenvironment-Mediated Drug Resistance in AML
Source: Cancers (Basel). 2023 Mar 27;15(7):1988. doi: 10.3390/cancers15071988 (PMC10093176; doi:10.3390/cancers15071988)

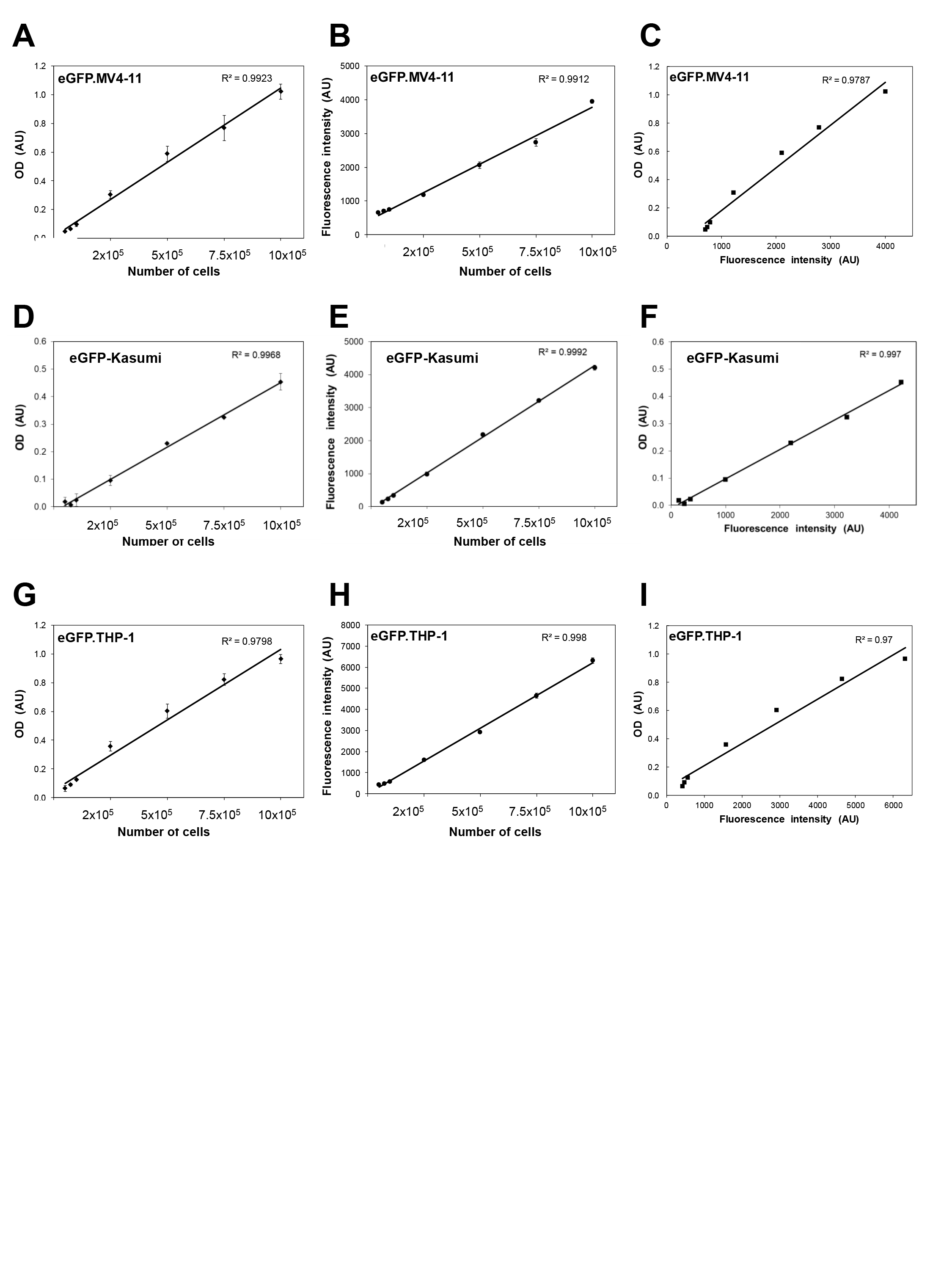

Supplement: Supplementary file 1 [file cancers-15-01988-s001.zip › Figure S1.tif]

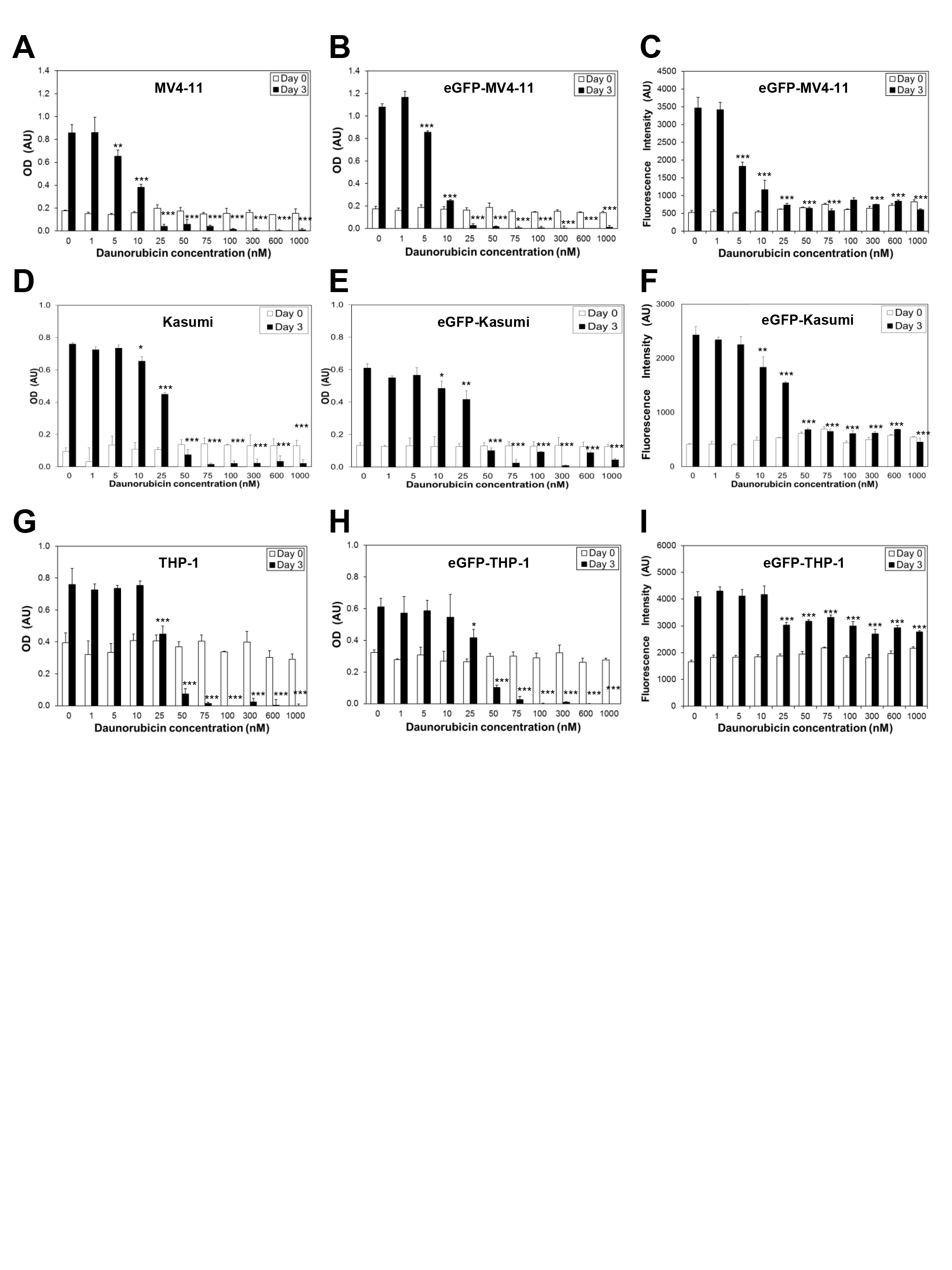

Supplement: Supplementary file 1 [file cancers-15-01988-s001.zip › Figure S2.tif]

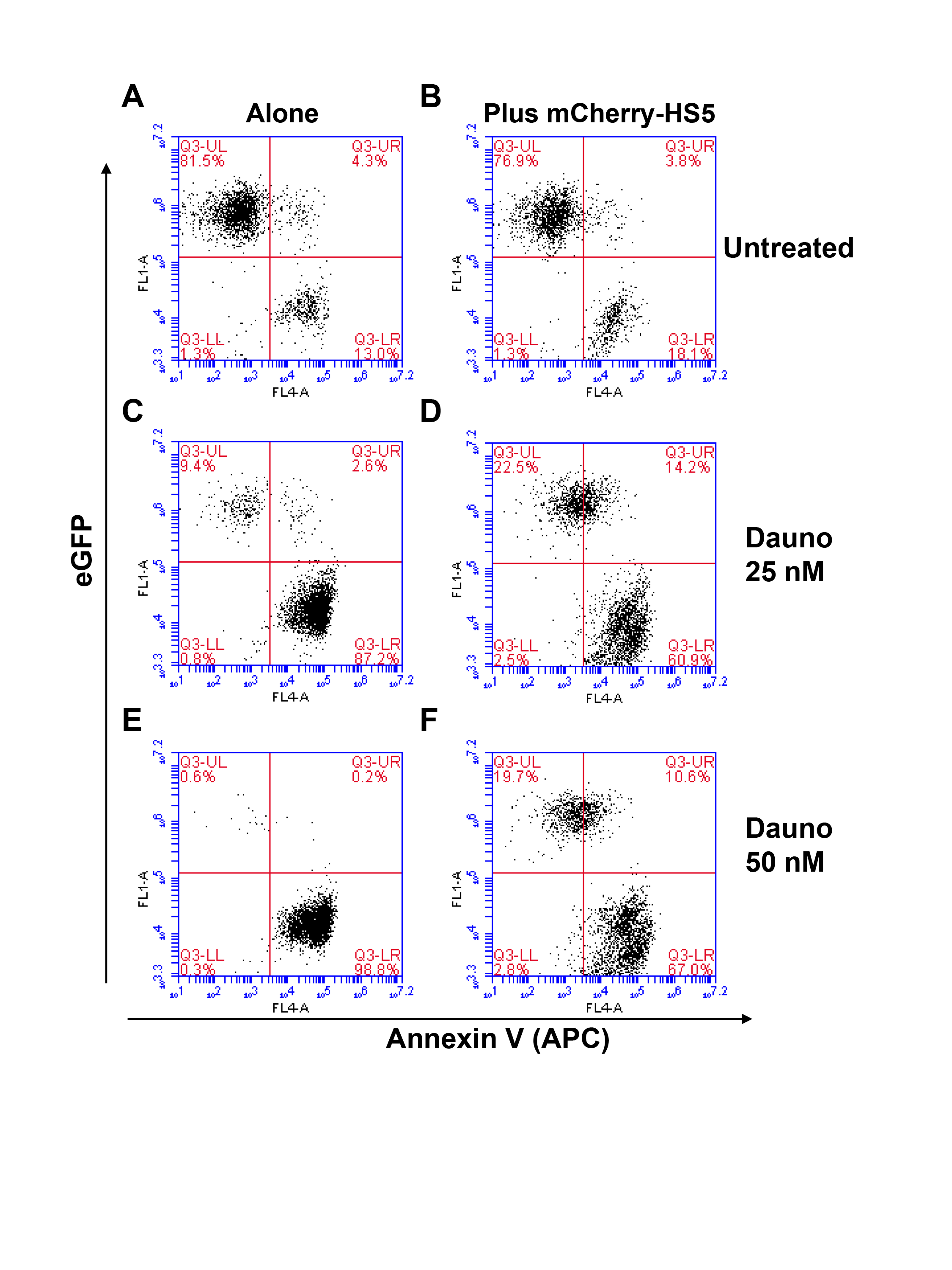

Supplement: Supplementary file 1 [file cancers-15-01988-s001.zip › Figure S3.tif]

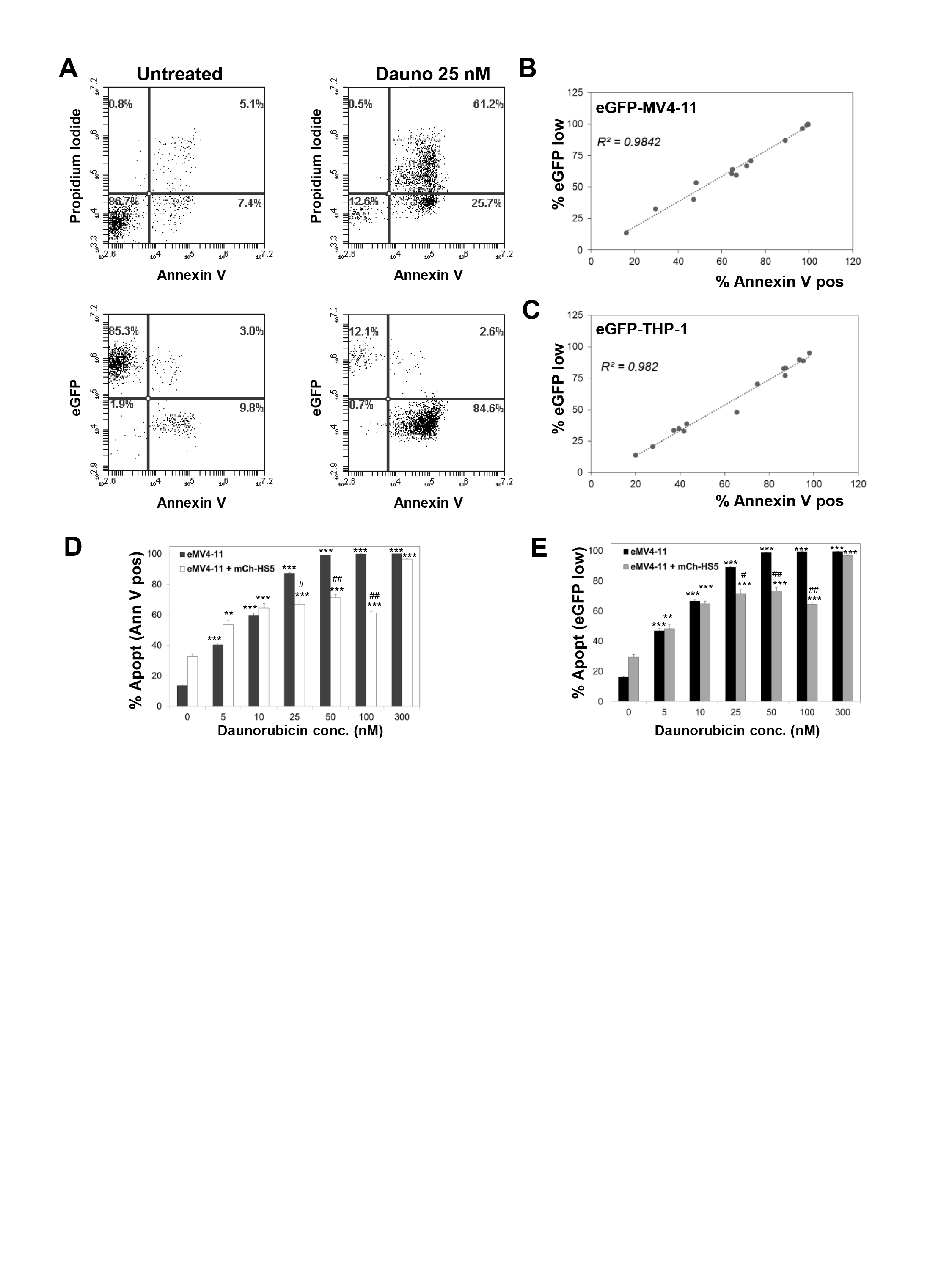

Supplement: Supplementary file 1 [file cancers-15-01988-s001.zip › Figure S4.tif]

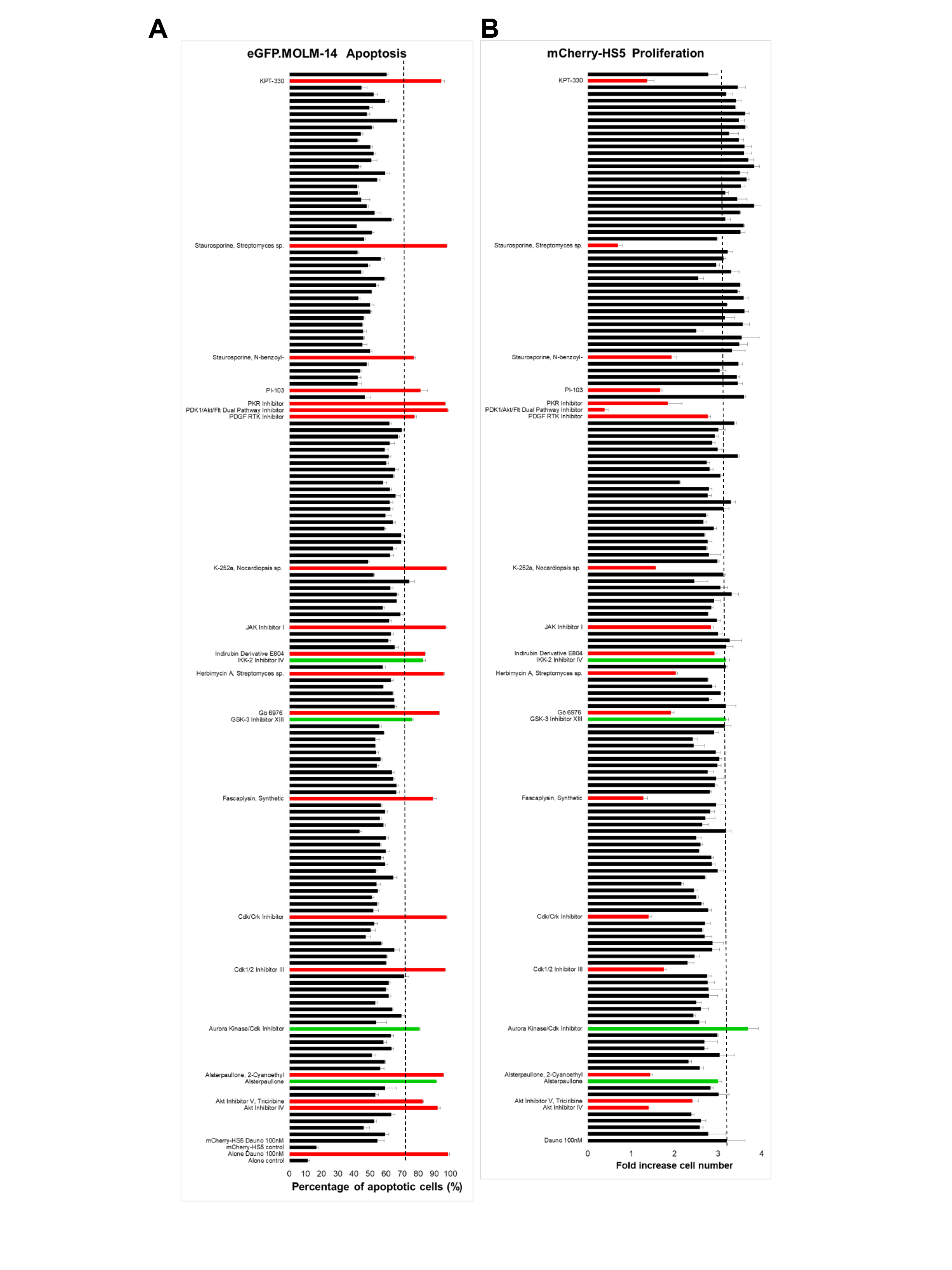

Supplement: Supplementary file 1 [file cancers-15-01988-s001.zip › Figure S5.tif]
